# Dysregulation of Neuropeptide and Tau Peptide Signatures in Human Alzheimer’s Disease Brain

**DOI:** 10.1021/acschemneuro.2c00222

**Published:** 2022-06-27

**Authors:** Sonia Podvin, Zhenze Jiang, Ben Boyarko, Leigh-Ana Rossitto, Anthony O’Donoghue, Robert A. Rissman, Vivian Hook

**Affiliations:** † Skaggs School of Pharmacy and Pharmaceutical Sciences, University of California, San Diego, La Jolla, California 92093, United States; ‡ Biomedical Sciences Graduate Program, University of California, San Diego, La Jolla, California 92093, United States; § Department of Neurosciences, University of California San Diego, La Jolla, California 92093, United States; ∥ Veterans Affairs San Diego Health System, La Jolla, California 92093, United States

**Keywords:** Alzheimer’s disease, proteomics, peptidomics, neuropeptides, tau, neurotransmission

## Abstract

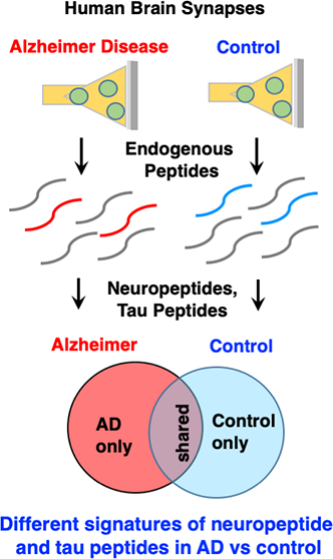

Synaptic dysfunction and loss occur in Alzheimer’s disease (AD) brains, which results in cognitive deficits and brain neurodegeneration. Neuropeptides comprise the major group of synaptic neurotransmitters in the nervous system. This study evaluated neuropeptide signatures that are hypothesized to differ in human AD brain compared to age-matched controls, achieved by global neuropeptidomics analysis of human brain cortex synaptosomes. Neuropeptidomics demonstrated distinct profiles of neuropeptides in AD compared to controls consisting of neuropeptides derived from chromogranin A (CHGA) and granins, VGF (nerve growth factor inducible), cholecystokinin, and others. The differential neuropeptide signatures indicated differences in proteolytic processing of their proneuropeptides. Analysis of cleavage sites showed that dibasic residues at the N-termini and C-termini of neuropeptides were the main sites for proneuropeptide processing, and data also showed that the AD group displayed differences in preferred residues adjacent to the cleavage sites. Notably, tau peptide signatures differed in the AD compared to age-matched control human brain cortex synaptosomes. Unique tau peptides were derived from the tau protein through proteolysis using similar and differential cleavage sites in the AD brain cortex compared to the control. Protease profiles differed in the AD compared to control, indicated by proteomics data. Overall, these results demonstrate that dysregulation of neuropeptides and tau peptides occurs in AD brain cortex synaptosomes compared to age-matched controls, involving differential cleavage site properties for proteolytic processing of precursor proteins. These dynamic changes in neuropeptides and tau peptide signatures may be associated with the severe cognitive deficits of AD.

## Introduction

Synaptic neurotransmitters are essential for cell–cell communication among neurons in neurological diseases and in health. Synaptic dysfunction and loss occur in brains of Alzheimer’s disease (AD), which leads to severe cognitive deficits and brain neurodegeneration.^1−5^ Neuropeptides comprise the major group of neurotransmitters,^6−9^ which work together with the classical small-molecule neurotransmitters numbering about 14.^10,11^ Neuropeptides consist of small peptides of approximately 3–40 amino acid residues in length that are generated from proneuropeptide precursors. The diverse amino acid sequences of neuropeptides define their functions in regulating brain behaviors including cognitive deficits of AD.^12−15^

Neurotransmission utilizes repertoires of neuropeptides to mediate communication among neurons and cells in the nervous system.^11^ The full spectrum of neuropeptide signatures is hypothesized to differ in human AD brain compared to age-matched controls. Global, unbiased neuropeptidomics mass spectrometry allows assessment of neuropeptide profiles. Therefore, the goal of this study was to investigate synaptic neuropeptide signatures by neuropeptidomics analysis of human AD brain cortex compared to age-matched controls. We examined human brain cortex in synaptosome preparations of nerve terminals where neurotransmitters are stored in secretory vesicles for release to mediate functional communication among target neurons. The results demonstrated distinct profiles of neuropeptides in AD compared to control groups.

Within the proneuropeptides, the neuropeptides are flanked largely by dibasic amino acid residues (Lys–Arg, Arg–Lys, Arg–Arg, Lys–Lys) that are predicted to be recognized and cleaved by proteases for neuropeptide production.^6−9^ However, direct analysis of neuropeptide sequences to elucidate proneuropeptide cleavage sites in human brain has not yet been achieved in the field. Extensive studies of non-human animal models have demonstrated processing at dibasic residues of proneuropeptides.^6−9^ Therefore, this study quantitated the frequencies of amino acid residues at the P1–P1′ cleavage sites as well as residues at the P4 to P4′ residues of the peptide sequences encompassing the cleavage sites of proneuropeptides. The results showed that human brain cortex from AD and controls primarily utilize dibasic residue cleavage sites at the N-termini and C-termini of neuropeptides for proteolysis of proneuropeptides to generate the identified neuropeptides. Significantly, the AD and control groups each displayed differences in the preferred residues adjacent to the cleavage sites in the P4 to P4′ sequence. These differential cleavage properties generated distinct signatures of neuropeptides in AD compared to control brain cortex synaptosomes.

Further significant findings indicated distinct profiles of tau peptides in the AD group compared to the age-matched control group of human brain cortex synaptosomes. Tau toxicity and pathogenesis in AD is a hallmark of this and related neurodegenerative diseases.^16−19^ These peptidomics data demonstrated unique tau peptides that were not previously identified in the field. These identified tau peptides were derived from the tau protein through proteolysis using similar and differential cleavage sites in the AD brain cortex compared to the control group.

Differential neuropeptide products derived from proneuropeptides may implicate differences in protease components expressed in AD brains compared to control. Analysis of synaptic protease systems by quantitative proteomics showed that differential profiles of proteases were present in the AD compared to control. Dysregulation of several proteases were observed, including several known to participate in the production of neuropeptides.

Overall, dysregulation of neuropeptides and tau peptides in AD brain cortex synaptosomes compared to age-matched controls was demonstrated by this study. These findings indicate that differential signatures of neuropeptides and tau peptides in human brain may be associated with the severe cognitive deficits of AD.

## Results

### Hypothesis and Experimental Workflow for Synaptic Peptidomics Analysis of Human AD and Control Brain Cortex

**Figure 1:**
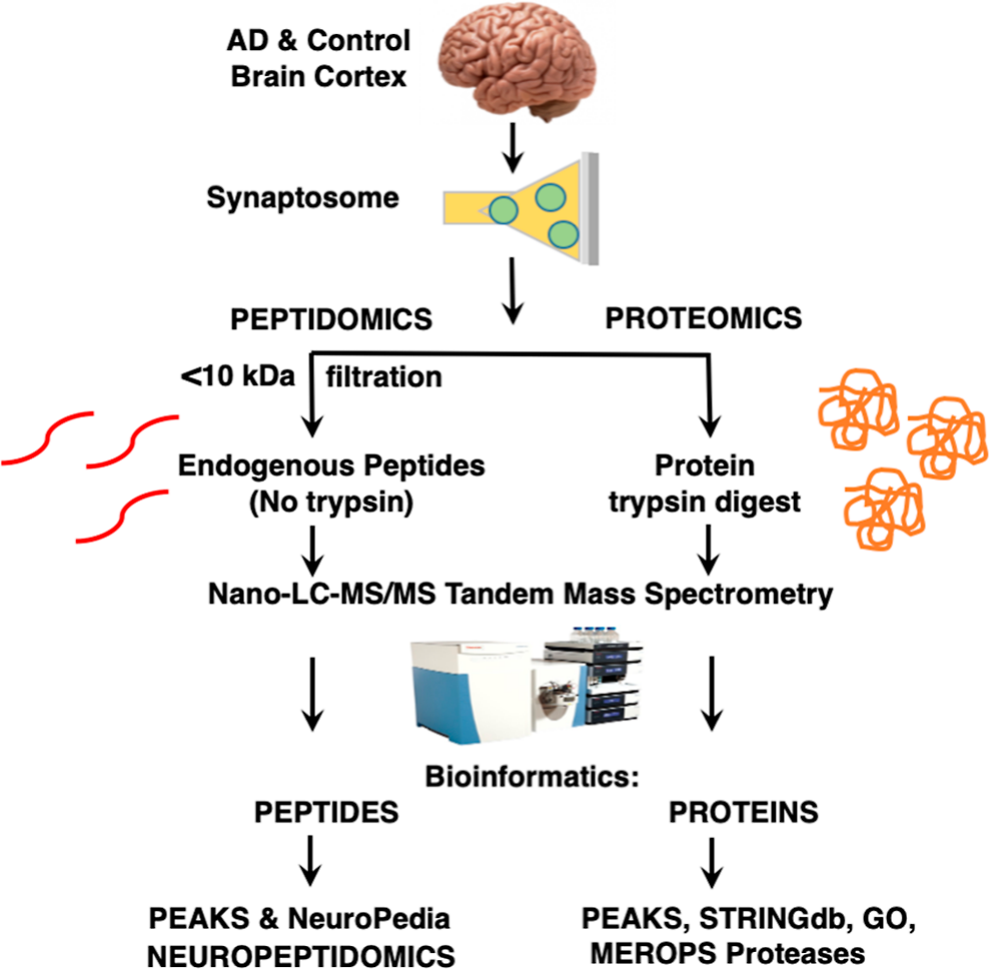
Workflow for neuropeptidomics and proteomics analyses of synaptosomes isolated from AD and age-matched control brain cortex. Brain cortex tissues from AD and age-matched controls were subjected to gentle homogenization and differential centrifugation for isolation of synaptic nerve terminals as synaptosome preparations. Low-MW peptides were obtained by filtration through a 10 kDa membrane for peptidomics analysis of endogenous peptides and neuropeptides, and retained proteins were digested with trypsin for proteomics analysis. After nano-LC–MS/MS tandem mass spectrometry of samples, bioinformatics by PEAKS provided identification and quantitation of peptides. Neuropeptides within the peptidomics data set were searched via the NeuroPedia database of neuropeptides to compile synaptic neuropeptidomics data. Proteomics data were assessed for protease components by searching the MEROPS protease database and evaluated for protein networks by STRINGdb and GO.

The project strategy was to assess the hypothesis for dysregulation of synaptic peptide neurotransmitters in AD compared to age-matched control human brain cortex. Synapses were isolated from frozen brain tissues as synaptosome preparations by differential centrifugation (Figure 1). Intact synaptosomes can be readily isolated from human frozen brain tissues.^20^ Synaptosome peptides and proteins were separated by filtration through a 10 kDa cutoff membrane with the flow through containing a low-molecular weight (MW) peptide pool. Endogenous peptides of the low-MW pool were subjected to peptidomics for identification and quantitation by nano-LC–MS/MS tandem mass spectrometry with bioinformatics using PEAKs and NeuroPedia. Proteomics was conducted by subjecting synaptosome proteins to trypsin digestion, followed by nano-LC–MS/MS combined with protein identification with label-free quantitation (LFQ) and functional analysis using bioinformatics tools of PEAKS, STRING, gene ontology (GO), and MEROPS protease analyses conducted as have been reported.^7,22^

It is noteworthy that functional synaptosomes isolated from postmortem human brain tissues retain functional neurotransmitter release, indicating the presence of intact transmitter secretory vesicles at the nerve terminals. The intact secretory vesicles containing neuropeptide and tau peptides result in protection of these peptides from lysosomal degradation. The integrity of synaptosomes isolated from frozen human brain tissues has been reported to be maintained for about a day postmortem time.^20,23^ Furthermore, rigorous analysis of data for this study required that for a peptide to be considered present in either the AD or control group, it must be present in at least three of the four biological replicate samples; this requirement may minimize particular peptides subject to degradation during the study. Overall, the synaptosome preparation provides a unique model of *in vivo* neurotransmitter signatures at synaptic nerve terminals of AD compared to control brains.

### Brain Tissues from Human AD Compared to Age-Matched Control Individuals Assessed for Cognitive Status

The statuses of cognitive dysfunctions of AD compared to age-matched subjects were measured by mini-mental state examination (MMSE).^24^ MMSE measures cognitive functions on a score range of 1–30 with normal cognitive functions represented by scores of 26–30 and cognitive deficits indicated by scores of 25 and less. Cognitive impairment of AD individuals is indicated by low MMSE scores of 1–18 (Table 1). Age-matched healthy controls had normal MMSE scores of 29–30 (Table 1). Brain tissues from AD and age-matched controls consisted of four samples for each group, composed of half male and half female (Table 1). The postmortem interval (PMI) time was recorded for brain samples (Table 1), and all samples were de-identified.

**Table 1:** AD and Age-Matched Control Human Brain Cortex Tissues^a^

diagnosis	age	sex	PMI (h)	MMSE score
normal	84	M	36	30
normal	94	M	12	30
normal	80	F	12	29
normal	83	F	72	29
AD	81	M	5	18
AD	89	M	10	15
AD	85	F	6	7
AD	77	F	6	1

a Brain cortex tissues were obtained from the Shiley-Marcos Alzheimer’s Disease Research Center (ADRC) Brain bank at UC San Diego. Tissue samples from normal controls and AD subjects are shown with respect to age, sex, PMI, and MMSE cognitive scores.

### Distinct and Shared Neuropeptides in Synaptosomes from Brain Cortex of AD Compared to Age-Matched Controls

**Figure 2:**
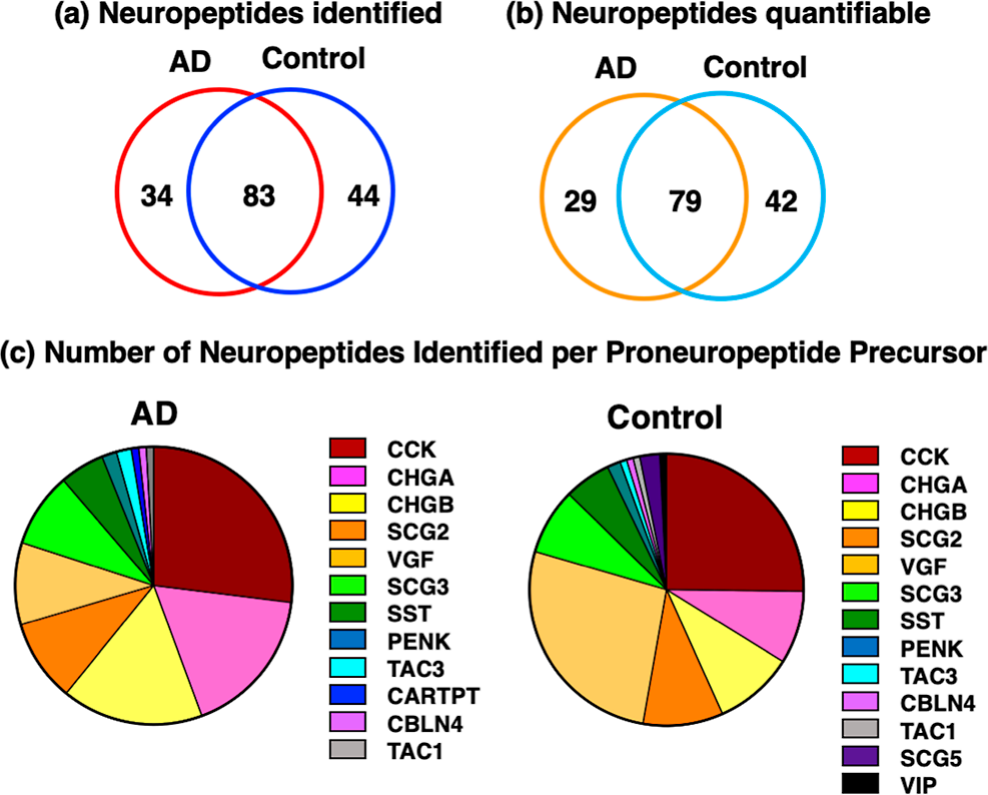
Distinct and shared neuropeptides in synaptosomes from AD and control brain cortex. (a) Identified neuropeptides. The unique neuropeptides identified in AD and control synaptosomes were compared with respect to the numbers of neuropeptides present only in the AD group, present in only the control group, and shared by both groups, as shown by the Venn diagram. (b) Identified and quantitated neuropeptides. The identified and quantitated neuropeptides in AD and control groups were compared with respect to the neuropeptides present in only AD or only in the control and shared neuropeptides present in both groups, as shown by the Venn diagram. (c) Neuropeptide precursors. The proneuropeptide precursors identified by proteomics data are illustrated with respect to relative abundance of peptides derived from each of the precursors. AD and control groups were compared by the illustrated pie charts.

Neuropeptides identified consist of peptide sequences included within known proneuropeptide precursors, according to the NeuroPedia database.^25^ Identification of neuropeptides revealed unique neuropeptides found in only the AD group (Figure 2a,b). Distinct neuropeptides were also found in only the control group (Figure 2a,b). A larger number of neuropeptides were present in both the AD and control groups (Figure 2a,b). The proneuropeptide precursors which generate the identified neuropeptides are shown as pie charts for the AD and control synaptosome groups (Figure 2c). Peptides derived from the main synaptosomal proneuropeptides of cholecystokinin (CCK), CHGA, CHGB, SCG2, VGF, and SCG3 (Figure 2c) are indicated for those only in AD, only in control, and shared in both AD and control groups (Table S1 and Figures S1–S5). Most of the synaptosomal neuropeptides are derived from CCK, CHGA, CHGB, SCG2, VGF, and SCG3 proneuropeptides (Figure 2c).

### Unique Chromogranin-Related Neuropeptides in AD Compared to Control Synaptosomes

**Figure 3:**
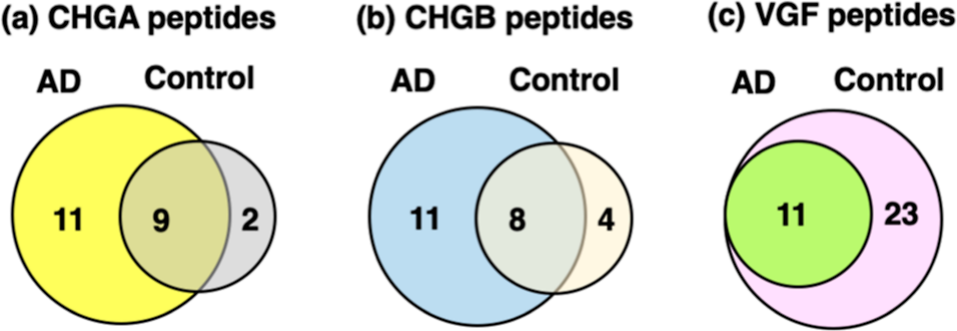
Peptides derived from chromogranin A (CHGA), chromogranin B (CHGB), and VGF (nerve growth factor inducible) in synaptosomes from AD compared to control brain cortex. Neuropeptides derived from the proneuropeptides of CHGA (panel a), CHGB (panel b), and VGF (panel c) are shown with respect to those identified in AD and control groups in Venn diagrams.

The chromogranin family of neuropeptides consists of those derived from the proneuropeptides chromogranin A (CHGA), chromogranin B (CHGB), secretogranin 2 (SCG2), and secretogranin 3 (SCG3). Numerous CHGA-derived and CHGB-derived neuropeptides (11 peptides each) were found in only AD synaptosomes and not in controls (Figure 3a,b). Several CHGA- and CHGB-derived peptides (two and four peptides, respectively) were identified in only control synaptosomes, indicating their absence in the AD group. Also, shared peptides were identified in both AD and control groups.

#### CHGA Neuropeptides

**Figure 4:**
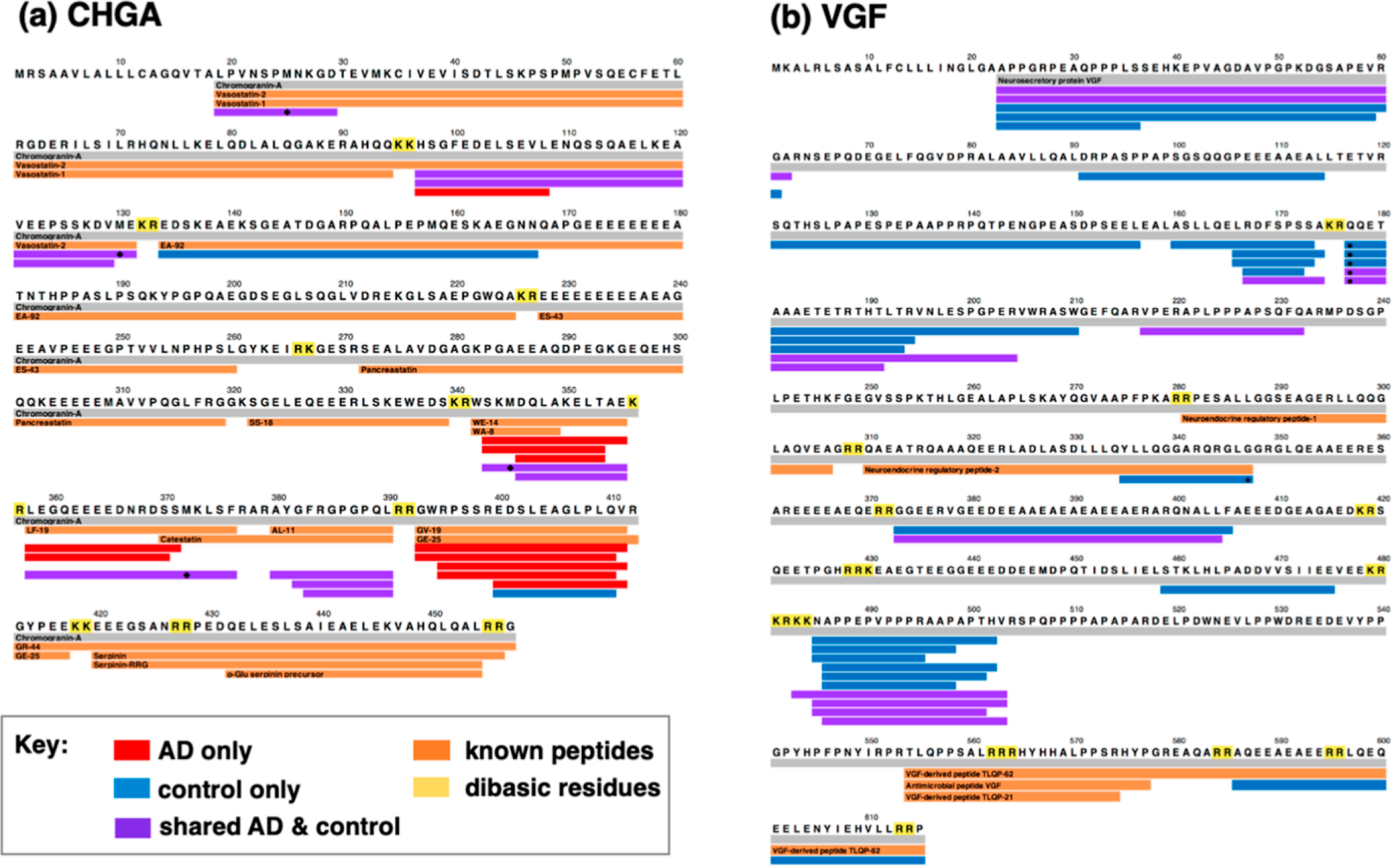
Mapping of peptides derived from CHGA and VGF in synaptosomes from AD compared to control brain cortex. (a) CHGA-derived neuropeptides. Peptide mapping shows the neuropeptides derived from the CHGA proneuropeptide. (b) VGF-derived neuropeptides. Peptide mapping shows the neuropeptides derived from the VGF proneuropeptide. For panels (a,b), the color-coded peptides indicate those present in only AD (red), present in only the control (blue), and shared by both AD and control synaptosomes (purple).

Mapping of neuropeptides derived from CHGA (Figure 4a) illustrates the different and similar peptide cleavage products in AD compared to control synaptosomes (Figure 4a). Peptides present in only AD synaptosomes consisted of peptides covering the WE-14, WA-8, LF-19, GV-19, and GE-25 peptide domains of CHGA. The control only peptides represented the EA-92 and GV-19/GE-25 domains of CHGA. The shared peptides identified in both AD and controls represented CHGA domains of vasostatin, WE-14, WA-8, LF-19, and catestatin. Significantly, differential processing of CHGA to diverse neuropeptides occurs in AD compared to control brain synaptosomes.

#### CHGB Neuropeptides

Peptides derived from the CHGB precursor were found in three categories of those present in only the AD group (not in controls) consisting of 11 peptides, present in only the control group (absent in AD) consisting of 4 peptides, and shared by both AD and controls consisting of 8 peptides (Figure 3b). Mapping of these peptides to the CHGB precursor (Figure S1) illustrates the different and similar cleavage products generated by CHGB proteolysis in AD and control brain synaptosomes.

#### Secretogranin Neuropeptides

Peptide products derived from SCG2 and SCG3 were largely similar in AD and control synaptosomes represented by 8 peptides out of a total of 10–11 (Table S1). However, peptide mapping of those derived from SCG2 and SCG3 shows several peptides unique to the AD group or control group (Figures S2, S3).

### VGF-Derived Peptides Absent in AD Compared to Control Synaptosomes

Out of a total of 34 peptides derived from VGF (known as VGF nerve growth factor inducible), more than half of the peptides (numbering 23) were absent in AD compared to controls (Figure 3c). Peptide mapping showed that peptides were present in only controls or shared by both AD and controls (Figure 4b).

These data show a significant loss of VGF peptides in AD compared to controls.

### Differential Cleavages Utilized for Neuropeptide Production in AD Compared to Controls

**Figure 5:**
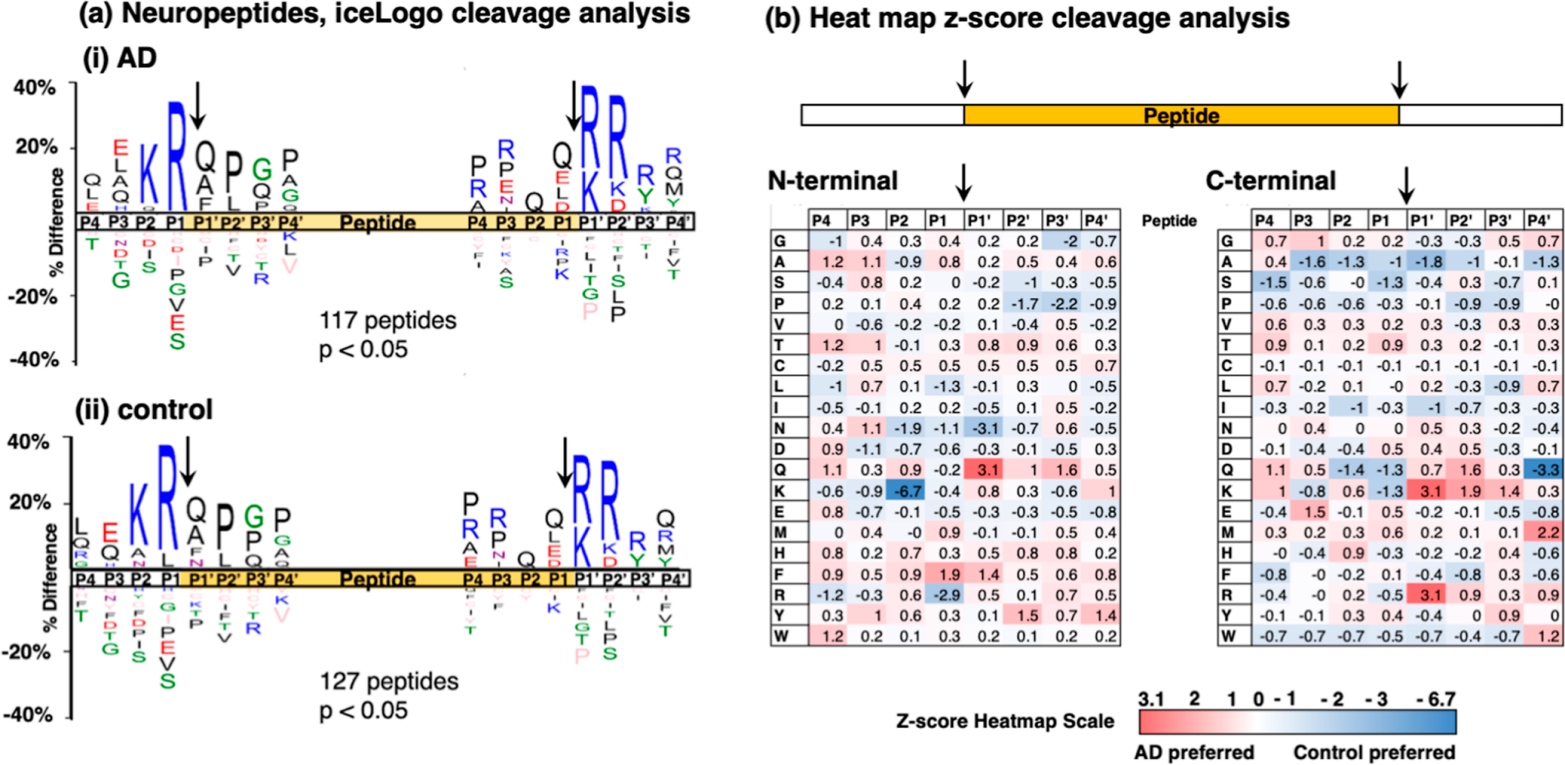
Cleavage site analysis for neuropeptide production from proneuropeptides in AD compared to control synaptosomes from brain cortex. (a) IceLogo analysis. IceLogo illustrates the relative frequencies of amino acids at the P4 to P4′ positions at N-terminal and C-terminal cleavages of neuropeptides within precursors that are utilized to generate neuropeptides in the AD (panel i) and control (panel ii) groups. Color codes for amino acids indicate acidic residues in red, basic residues in blue, polar residues in green, nonpolar residues in black, asparagine in purple, and residues never found in pink. (b) Heatmap comparison of preferred residues at N-termini and C-termini of neuropeptides within the proneuropeptide precursors. The heatmap illustrates *z*-scores (calculated as explained in the methods) that indicate the frequency of amino acid residues at P4 to P4′ positions of cleavage sites at N-termini and C-termini within proneuropeptides of neuropeptides in AD compared to control synaptosomes.

IceLogo analysis^26^ of cleavages occurring within proneuropeptides to generate the identified neuropeptides in AD and control synaptosomes calculated the frequency of amino acid residues at the P1–↓P1′ cleavage site and at residues adjacent to the cleavage site at positions P4 to P4′ (Figure 5). The majority of cleavages occurred at dibasic residue sites represented primarily by Lys–Arg (K–R) at the N-termini of neuropeptides and by R/K–R/K at the C-termini of neuropeptides, observed for neuropeptides in both AD and control synaptosomes (Figure 5a).

More detailed analysis of cleavage site frequencies was gained by quantitative assessment of *z*-scores of AD compared to control cleavage preferences illustrated in heatmaps (Figure 5b). For cleavages at the N-termini of neuropeptides, the control group displayed clear preference for Lys and Arg at the P2 and P1 residues, respectively. The AD group displayed greater preference for Gln as the P1′ residue. For cleavages at the C-termini of neuropeptides, Lys and Arg were the preferred residues at the P1′ position for the AD group. These data illustrate similarities and differences in cleavage sites for neuropeptide production in the AD compared to control synaptosomes.

### Differential CHGA Cleavages in AD Compared to Controls

**Figure 6:**
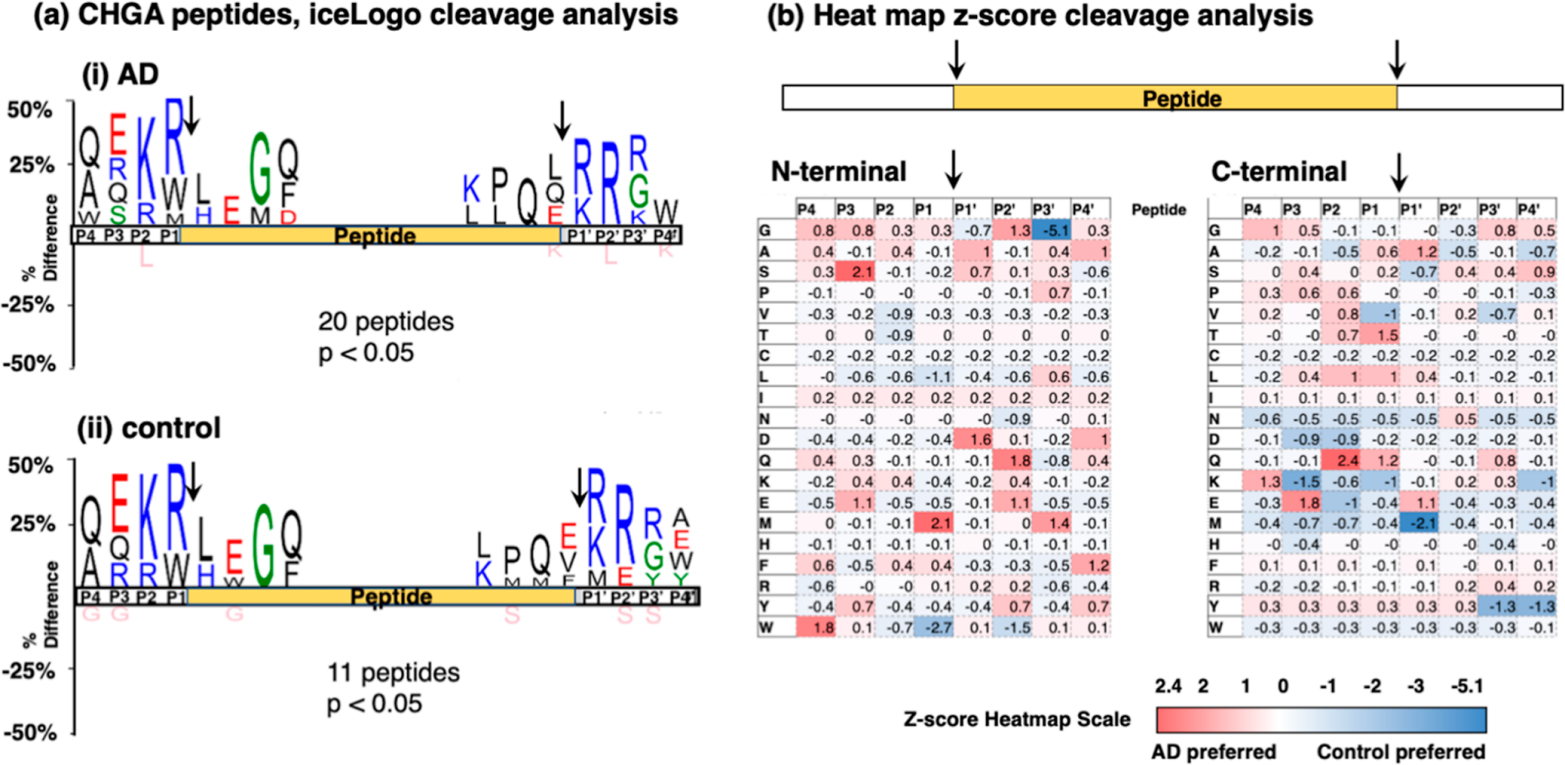
Cleavage site analysis of CHGA-derived neuropeptides in AD compared to control brain synaptosomes. (a) IceLogo analysis. IceLogo shows the relative frequencies of residues at the P4 to P4′ positions of N- and C-terminal cleavages of neuropeptides derived from CHGA in AD (panel i) and control (panel ii) synaptosomes. Color codes for amino acids indicate acidic residues in red, basic residues in blue, polar residues in green, nonpolar residues in black, asparagine in purple, and residues never found in pink. (b) Heatmap comparison of preferred residues at N- and C-termini of neuropeptides within CHGA. The heatmap shows *z*-scores that indicate the frequency of amino acid residues at P4 to P4′ positions of cleavage sites at N-termini and C-termini of neuropeptides within CHGA.

For CHGA-derived peptides, CHGA cleavage site analysis in AD and controls showed similar cleavage preferences for K–R at the N-termini of neuropeptides, and R/K–R/K at the C-termini of neuropeptides shown by IceLogo (Figure 6a). Heatmaps of *z*-scores for the relative preferences of residues at the N-termini and C-termini in the AD and control groups showed differences (Figure 6b).

At N-terminal peptide cleavage sites (Figure 6b), the AD group showed amino acid (indicated by single letter codes) preferences (*z*-scores of >0.9) at P1 to P4 residues of (a) M at P1, (b) no main preferences at P2, (c) S and E at P3, and (d) W at P4, and preferences at P1′ to P4′ were (a) A and D at P1′, (b) G, Q, and E at P2′, (c) M at P3′, and (d) A, D, and F at P4′. In contrast, the control group N-terminal cleavages displayed preferences at P1–P4 of (a) L and W at P1, (b) V and T at P2, and (c) no main preferences at P3 or P4, and preferences at P1′ to P4′ were (a) no main preferences at P1′, (b) N and W at P2′, (c) G (strong preference) at P3′, and (d) no preferences at P4′.

At C-terminal peptide cleavage sites (Figure 6b), the AD neuropeptide cleavages showed preferences at P1 to P4 positions of (a) T and Q at P1, (b) L and Q at P2, (c) E at P3, and (d) G and K at P4, and preferences at P1′ to P4′ were (a) A and E at P1′, (b) no major preferences at P2′, (c) no major preferences at P3′, and (d) S at P4′. In contrast, the control C-terminal cleavages showed preferences at P1–P4 of (a) V and K at P1, (b) D and E at P2, (c) D and K at P3, and (d) no major preference at P4, and preferences at P1′ to P4′ were (a) M at P1′, (b) no main preference at P2′, (c) Y at P3′, and (d) K and Y at P4′.

Overall, these data show that dibasic residue sites are the primary locations of CHGA cleavages and that variant amino acid preferences occur at adjacent neighboring residues for the AD neuropeptides compared to the control neuropeptides.

### Differential VGF Cleavages for Peptide Production in AD and Controls

**Figure 7:**
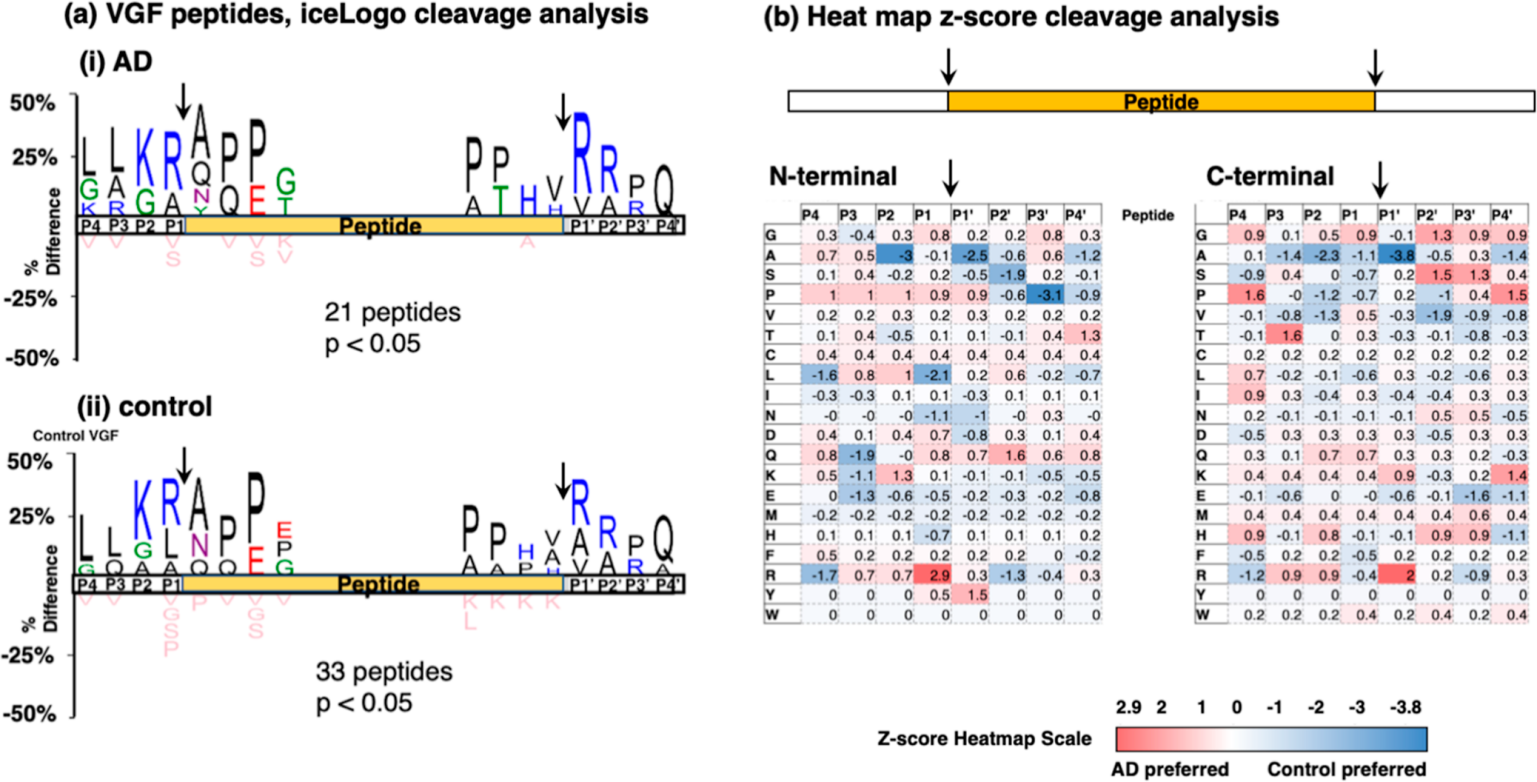
VGF peptide cleavage site analysis in AD compared to controls. (a) IceLogo analysis. IceLogo shows the relative frequencies of residues at the P4 to P4′ positions of N- and C-terminal cleavages of neuropeptides derived from VGF in AD (panel i) and control (panel ii) synaptosomes. Color codes for amino acids indicate acidic residues in red, basic residues in blue, polar residues in green, nonpolar residues in black, asparagine in purple, and residues never found in pink. (b) Heatmap comparison of preferred residues at N- and C-termini of neuropeptides within VGF. The heatmap shows *z*-scores that indicate the frequency of amino acid residues at P4 to P4′ positions of cleavage sites at N-termini and C-termini of neuropeptides within VGF.

IceLogo cleavage site analysis of AD and control peptides derived from VGF showed Lys–Arg as the major residues at the N-terminal side of peptide products and showed Arg–Arg as the main residues at the C-terminal side (Figure 7a). Also, the relative frequencies of the primary residues located at P4 to P4′ of N- and C-termini of VGF-derived peptides were similar for the AD and control groups.

Quantitative assessment of preferred residues at P4 to P4′ of N- and C-terminal cleavages by *z*-scores shown by heatmaps illustrates differential patterns of cleavages to generate peptides identified in AD compared to control synaptosomes (Figure 7b). At the N-termini, the AD group showed preferences at P1 to P4 residues of (a) P at P1, (b) P, L, and K at P2, (c) P at P3, and (d) P at P4, and preferences at P1′ to P4′ residues were (a) P and Y at P1′, (b) Q at P2′, (c) no preferences at P3′, and (d) T at P4′. The control group showed some differential preferences for VGF cleavage site sequences represented by P1 to P4 residues of (a) L and N at P1, (b) A (strong preference) at P2, (c) Q, K, and E at P3, and (d) L and R at P4, combined with P1′ to P4′ residues of (a) A and N at P1′, (b) S and R at P2′, (c) P (strong preference) at P3′, and (d) A and P at P4′.

At C-termini, the AD group showed preferences at P1 to P4 residues of (a) G at P1, (b) R at P2, (c) T and R at P3, and (d) G, P, I, and H at P4, and preferences at P1′ to P4′ residues were (a) K and R at P1′, (b) G, S, and H at P2′, (c) G, S, and H at P3′, and (d) G, P, and K at P4′. The control group showed some different preferences for VGF cleavage site sequences represented by P1 to P4 residues of (a) A at P1, (b) A, P, and V at P2, (c) A at P3, and (d) S and R at P4, combined with P1′ to P4′ residues of (a) A (strong preference) at P1′, (b) V at P2′, (c) V, E, and R at P3′, and (d) A, E, and H at P4′.

These findings show that dibasic Lys and Arg represent the main residues flanking the N- and C-termini of cleaved peptides within VGF for both the AD and control groups. However, AD and controls showed differences in adjacent residues at P4 to P4′ positions of the P1–↓P1′ cleavage sites utilized to generate VGF peptides.

### Distinct Tau Peptides in AD Compared to Control Brain Synaptosomes

**Figure 8:**
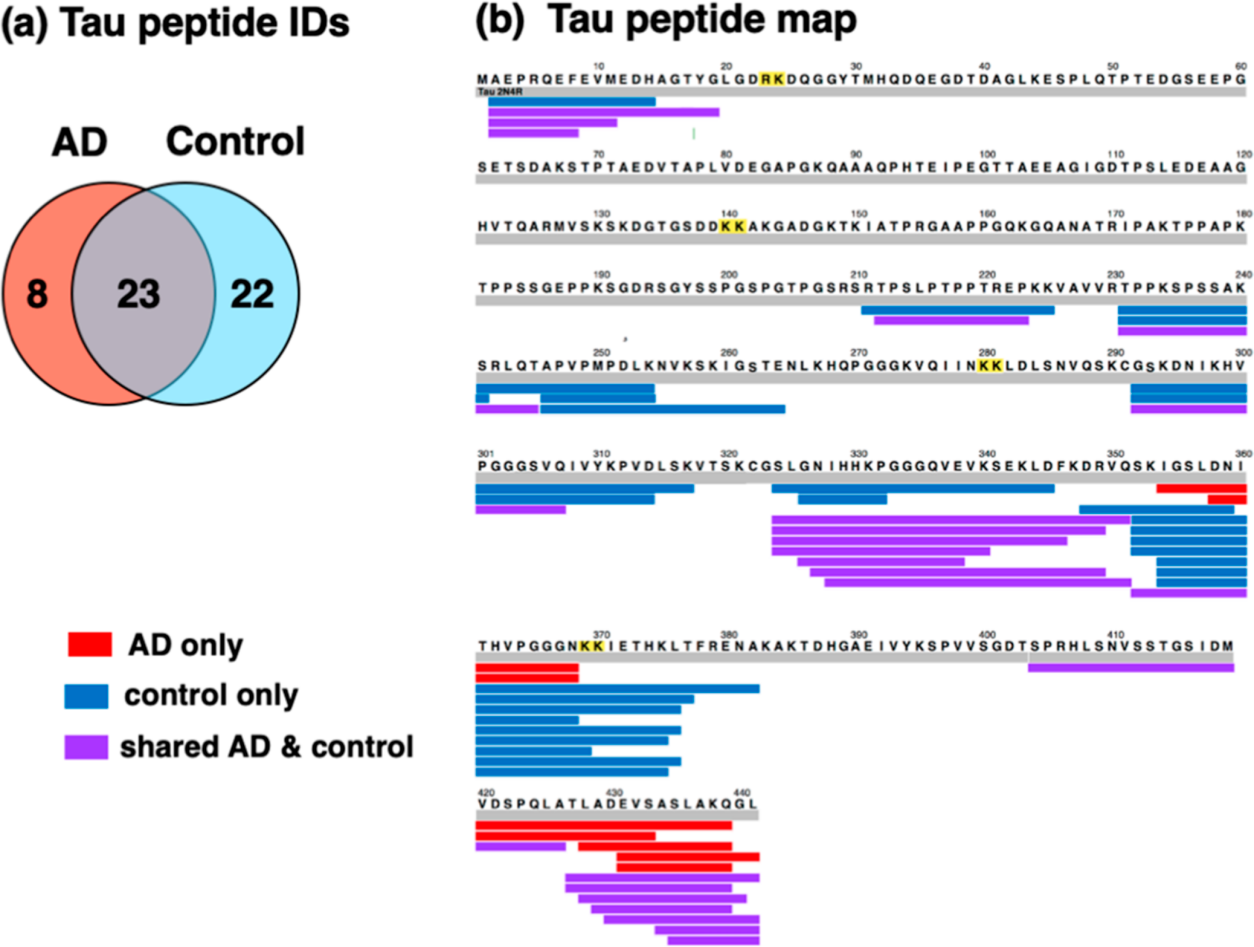
Distinct and shared tau peptides in synaptosomes from AD and control brain cortex. (a) Tau peptides identified in AD and control synaptosomes. The Venn diagram of the tau peptides identified in AD and control synaptosomes illustrates tau peptides present in only AD, only in the control, and shared in both AD and control groups. (b) Tau peptide mapping derived from the tau protein. Mapping of tau peptides derived from the tau protein are shown for those present only in AD (red), present only in the control (blue), and shared in both AD and control groups (purple).

Different profiles of low-MW tau peptides were identified in AD compared to control brain cortex synaptosomes (Figure 8) by peptidomics data (Figure S6). Among 53 tau peptides identified, 8 were unique to AD (Figure 8a). Furthermore, 22 tau peptides were absent in AD, shown by their presence in only the control group. In addition, 23 tau peptides were shared by the AD and control synaptosomes.

Peptide mapping showed that in AD synaptosomes, peptides were derived from the C-terminal domain of the tau protein (Figure 8b). However, tau peptides in the control group were derived from multiple regions of the tau protein, consisting of the N-terminal region, mid-region, and C-domain regions of tau.

### Differential Tau Protein Cleavages for Production of Synaptosomal Tau Peptides in AD Compared to Controls

**Figure 9:**
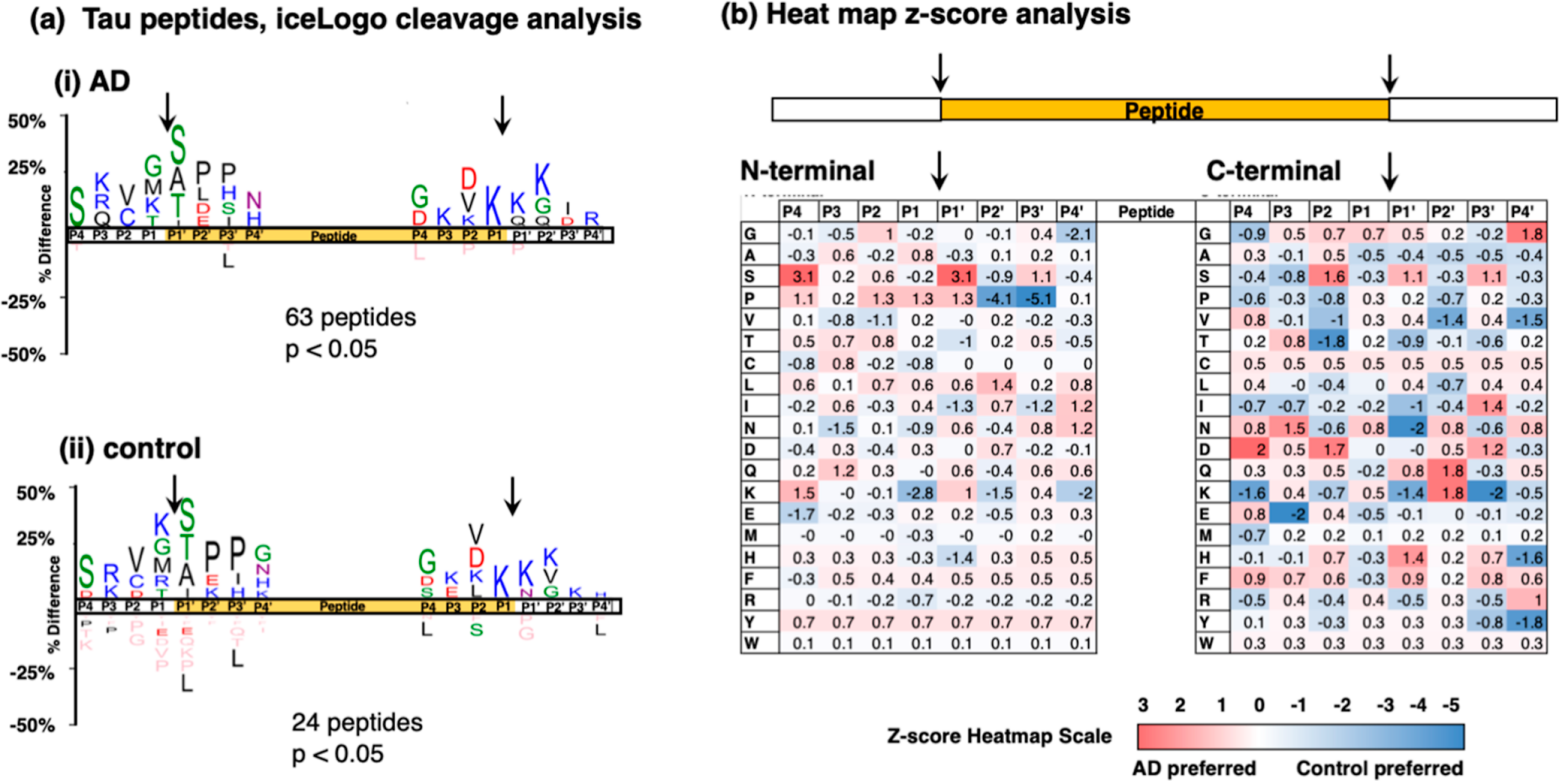
Cleavage site analysis of tau peptides derived from tau protein in AD compared to controls. (a) IceLogo analysis. IceLogo shows the relative frequencies of residues at the P4 to P4′ positions of N- and C-terminal cleavages of neuropeptides derived from the tau protein in AD (panel i) and control (panel ii) synaptosomes. Color codes for amino acids indicate acidic residues in red, basic residues in blue, polar residues in green, nonpolar residues in black, asparagine in purple, and residues never found in pink. (b) Heatmap comparison of preferred residues at N- and C-termini of neuropeptides within the tau protein. The heatmap shows *z*-scores that indicate the frequency of amino acid residues at P4 to P4′ positions of cleavage sites at N-termini and C-termini of tau peptides derived from the tau protein.

Cleavage site analysis of peptides derived from the tau protein showed that while the preferred amino acids at the P4 to P4′ positions of the P1–↓P1′ cleavage site were similar as illustrated by iceLogo assessment (Figure 9a), more detailed analysis by *z*-scores showed differences in the frequencies of residues adjacent to the tau cleavage sites in AD compared to controls (Figure 9b). IceLogo showed that cleavages for generation of N-termini of tau peptides in AD and controls utilized G/M/K–↓S/A/T as the primary residues at P1–↓P1′. Cleavages for generation of C-termini of tau peptides in AD and controls utilized K–↓K as the main residues at P1–↓P1′ (Figure 9a).

Significantly, differences in the frequencies of residues at P4 to P4′ positions of tau protein cleavage sites were observed for tau peptides present in AD compared to control brain synaptosomes, shown by *z*-scores illustrated in heatmaps (Figure 9b). For cleavages at N-termini of tau peptides, the AD group showed preferences (*z*-scores of at least 0.9) at P1 to P4 residues of (a) P at P1, (b) G and P at P2, (c) Q at P3, and (d) S (strongly preferred) and K at P4, and preferences at P1′ to P4′ were (a) S (strongly preferred), P, and K at P1′, (b) L at P2′, (c) S at P3′, and (d) I and N at P4′. In contrast, the control group cleavages at the N-termini of tau peptides displayed P1 to P4 preferences of (a) N and K (strongly preferred) at P1, (b) V at P2, (c) N at P3, and (d) E at P4, and preferences at P1′ to P4′ were (a) T, I, and H at P1′, (b) S, P (strongly preferred), and K at P2′, (c) P (strongly preferred) and I at P3′, and (d) G and K at P4′.

At the C-termini of tau peptides, the AD neuropeptide cleavages showed preferences at P1 to P4 consisting of (a) G and N as moderately preferred residues at P1, (b) S and D at P2, (c) N at P3, and (d) D and F at P4, and preferences at P1′ to P4′ were (a) S, H, and F at P1′, (b) Q and K at P2′, (c) S, I, and D at P3′, and (d) G and R at P4′ (Figure 9b). In contrast, the control group cleavages at the C-termini of tau peptides displayed P1 to P4 preferences of (a) no major preferred residues at P1, (b) V and T at P2, (c) E at P3, and (d) G and K at P4, and preferences at P1′ to P4′ were (a) T, I, N, and K at P1′, (b) V at P2′, (c) K at P3′, and (d) V, H, and Y at P4′.

Overall, these data show that tau peptides are generated by cleavage of the tau protein at basic and non-basic residues at P1–↓P1′ in the AD and control groups. However, differential variant amino acid preferences occur at P4 to P4′ residues of tau protein cleavage sites in the AD compared to the control group. Variant cleavages of the tau protein are consistent with the distinct profile of tau synaptosomal peptides identified in AD compared to controls.

### Distinct Proteomes in AD Compared to Control Brain Synaptosomes

**Figure 10:**
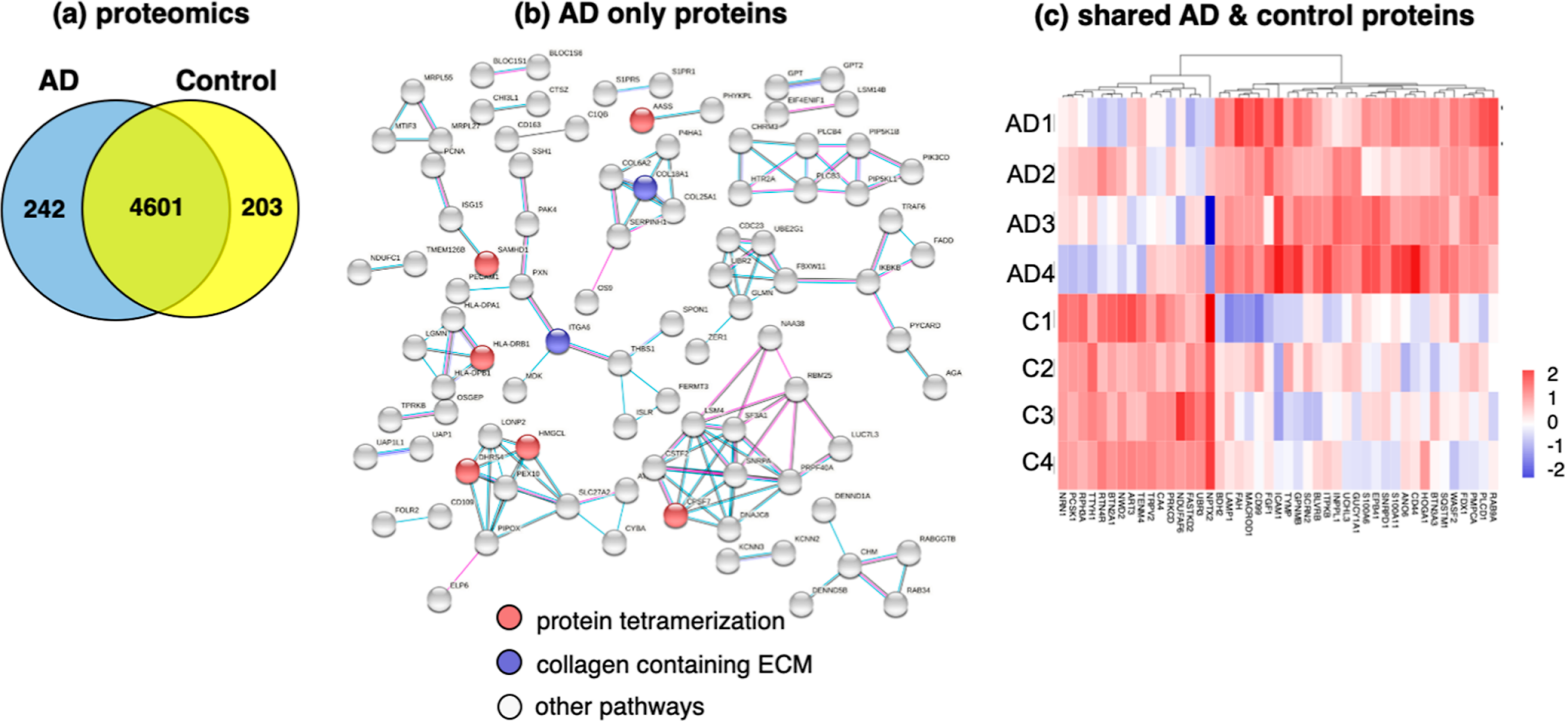
Proteomics reveals distinct and shared proteins in synaptosomes from AD compared to control brain cortex. (a) Proteins identified in AD and control synaptosomes. The Venn diagram shows proteins present in only AD, present in only the control, and shared by both AD and control synaptosomes. (b) Protein interaction network analysis of proteins present only in AD synaptosomes. Proteins present in only the AD group were assessed for predicted protein interaction networks evaluated by STRINGdb and GO. (c) Proteins shared by AD and control synaptosomes display upregulation and downregulation. Quantitation of the shared proteins was assessed for the ratio of log_2_(AD/control with significance of *p* < 0.05) to compare protein levels in the AD compared to control group. Upregulated proteins in AD are shown in red (compared to the control), and downregulated proteins are shown in blue (compared to the control).

To gain an understanding of the synaptosomal protein systems for neurotransmission, proteomics analyses were conducted and found distinct and shared proteins for AD and controls (Figure 10). Among the 5046 proteins identified for both groups, the AD group consisted of 242 proteins present only in AD, and the control group contained 203 proteins present in only the control (Figure 10a). Shared proteins present in both groups, numbering 4601, comprised most of the proteome data.

Protein functions were assessed by GO^27^ and STRING network^28,29^ analyses. For the AD only proteins, STRING interactions predicted protein interaction networks represented by GO functions of protein tetramerization and collagen related to the extracellular matrix (Figure 10b). For the control only proteins, no significant GO terms for the biological process or molecular function were found. For the shared proteins present in AD and control synaptosomes, quantitative evaluation indicated upregulation and downregulation of proteins in the AD group compared to the controls (Figure 10c).

### Differential Protease Expression in AD Compared to Control Brain Synaptosomes

**Figure 11:**
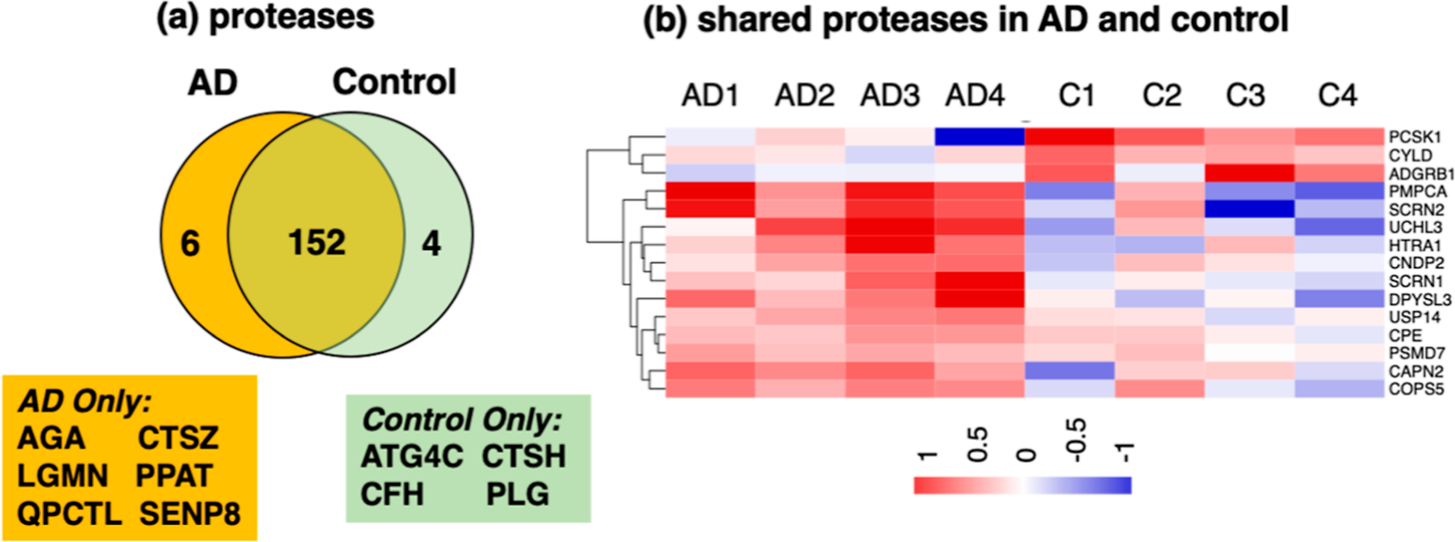
Proteases in synaptosomes from AD compared to control brain cortex. (a) Proteases in AD and control synaptosomes. Protease components of proteomics data were assessed by search of the protease MEROPS database.^30^ Comparison of proteases in AD and control groups is illustrated by the Venn diagram which shows proteases present in only the AD group, present in only the control group, and shared by both AD and control synaptosomes. (b) Upregulated and downregulated proteases in AD compared to controls. Quantitation of the shared proteases was assessed for the ratio of log_2_(AD/control with significance of *p* < 0.05) to compare protease levels in the AD and control groups. Upregulated proteins in AD are shown in red (compared to the control), and downregulated proteins are shown in blue (compared to the control).

Evaluation of the protease components of the synaptosomes (using the MEROPS protease database^30^) showed that distinct proteases in the AD group were present, absent, or upregulated and downregulated compared to the control group of synaptosomes (Figure 11). Among the identified proteases known to be involved in proneuropeptide processing,^7,31−34^ PCSK1 (proprotein convertase 1) was upregulated, carboxypeptidase E (CPE) was downregulated, and CTSH (cathepsin H) was absent in AD compared to control groups. This finding of differential regulation of several proneuropeptide processing proteases is consistent with observed differences in neuropeptide profiles in AD compared to control brain synaptosomes.

The tau protein cleavage sites differed from that of the proneuropeptides since the observed tau cleavages occurred at both basic and non-basic residues (Figure 9). Proneuropeptide processing utilized primarily basic residues at the cleavage sites (Figure 5). It is, therefore, of interest that several proteases were present in only the AD group (AGA, CTSZ, LGMN, PPAT, QPCTL, and SENP8), absent in the AD group (ATG4C, CTSH, CFH, and PLG), or upregulated and downregulated in the AD compared to control synaptosomes (Figure 11).

Overall, dysregulation of synaptosomal proteases is consistent with the differential signatures of neuropeptides and tau peptides observed in AD compared to control brain synaptosomes.

## Discussion

This study shows that neuropeptide transmitter dysregulation occurs as a significant feature of synaptic loss known to occur in brains of patients with AD that are cognitively impaired compared to age-matched controls. Neuropeptides represent the major group of neurotransmitters that are required for cell–cell communication in the brain. Diverse neuropeptides are generated from proneuropeptide precursors by proteolytic processing to generate thousands of synaptic neuropeptides. Global, unbiased profiling by neuropeptidomics utilized in this study showed that substantial dysregulation of synaptic neuropeptides occurred, illustrated by neuropeptides present only in the AD group, those that were absent in the AD group (present only in the control group), and shared neuropeptides present in both AD and age-matched control synaptosomes isolated from brain cortex. Significantly, dysregulated neuropeptides were derived from the proneuropeptides of chromogranin A (CHGA), chromogranin B (CHGB), and VGF (VGF nerve growth factor inducible). Further significant findings indicate unique synaptic tau peptides present or absent in the AD group, combined with tau peptides shared by both AD and control groups. These data demonstrate dysregulation of endogenous neuropeptide and tau peptides in human AD brain cortex synaptic locations necessary for cell–cell communication.

Among neuropeptides, CHGA is upregulated in AD and activates microglia to generate proinflammatory cytokines, which is consistent with inflammation in AD.^35−37^ Further, CHGA is present in amyloid plaques associated with accumulation amyloid-β.^38,39^ CHGB-derived neuropeptides are also co-localized with amyloid plaques in AD brains.^39^ The precise functions of CHGB neuropeptides are largely unknown at the present time. Altered neuropeptides derived from SCG2 (chromogranin C) and SCG3 (secretogranin 3) were also observed in AD compared to control synaptosomes. Neuropeptides derived from VGF (nerve growth factor inducible) regulate the neuronal firing rate,^40,41^ and VGF is upregulated during memory and learning activities.^40,42^ VGF gene expression is decreased in AD brains,^43^ and overexpression of VGF in the 5xFAD mouse model of AD results in improved memory functions;^44^ these findings indicate a role for VGF in memory functions that is compromised in AD. The spectrum of CCK and somatostatin neuropeptides was also altered in AD compared to control brain cortex synaptosomes. The results from this study show that synaptic changes in the molecular spectrum of neuropeptides derived from CHGA, CHGB, SCG2, SCG3, VGF, CCK, and somatostatin occur in human AD brain cortex compared to age-matched controls.

Tau accumulates in AD and induces neurotoxicity,^16−19^ and therefore, it is of interest that numerous synaptic tau peptides derived from the tau protein were identified in AD and control brain cortex. Different signatures of tau peptides were found in AD with those present only in AD or only in controls. Tau peptides present in both AD and control synaptosomes from brain cortex were also found. Previous studies have identified large tau fragments up to 35 kDa^19,45−49^ but have not reported profiles of small tau peptides. Thus, the small tau peptides of ∼10–30 amino acids identified in this study are new to the field. Notably, differential processing of the tau protein results in differential signatures of synaptic tau peptides in AD compared to controls.

Proteolytic processing generates neuropeptides from their larger proneuropeptide precursors.^31−34^ To gain an understanding of the amino acid residues at the precursor cleavage sites utilized to generate these peptides, the relative frequencies of residues at the P1–↓P1′ cleavage site and at neighboring P4 to P4′ residues were assessed by iceLogo.^26^ Proneuropeptide cleavages occurred primarily at dibasic residues sites (Lys–Arg, Arg–Lys, Arg–Arg, and Lys–Lys) flanking the N-termini and C-termini of neuropeptides. Significantly, this is the first report of the dibasic preference of proneuropeptide cleavages in human brain to the best of our knowledge. Prior studies have largely investigated non-human animal models for characterization of dibasic residue processing sites for production of neuropeptides.^31−34^ Significantly, our further evaluation of the human brain neuropeptides with respect to residues at P4 to P4′ residues adjacent to the P1–↓P1′ cleavage site showed differences in neuropeptides in the AD compared to the controls, indicated by *z*-scores. Differences in preferred residues at P4 to P2 and P2′ to P4′ between the AD and controls represent the different neuropeptides generated in the human brain in AD compared to controls. These cleavage properties predict that processing involves proteases that recognize and cleave dibasic residues at cleavage sites as well as adjacent, neighboring residues. Proteases are known to recognize both cleavage site and neighboring residues in a selective manner.^50−54^

Analysis of tau protein cleavage sites showed abundant utilization of Lys–↓Lys at the C-termini of tau peptides in AD and control groups. However, different residues were utilized at the N-termini of tau peptides, which consisted of G/M/K–↓S/A/T for the AD group and K/G/M/R–↓S/T/A for the control group. These data indicate similarities and differences between the AD and control N-terminal cleavage sites for production of tau peptides. Furthermore, neighboring residues of tau protein cleavage sites showed differences as well as similarities. These findings of different preferences for peptide sequence properties at cleavage sites of the tau protein illustrate the differential proteolytic processing mechanisms occurring for production of synaptic tau peptides in AD and control brain cortex.

To gain an understanding of protease systems present in AD and control groups, the spectrum of proteases present in AD and controls was assessed by proteomics data. The results showed dynamic protease changes shown by (1) proteases present only in the AD group, (2) proteases absent in the AD group, that is, present in only the control group, and (3) proteases that were upregulated and downregulated in the AD compared to the control group. Several proteases known to participate in neuropeptide production^31−34^ were dysregulated, which consisted of PCSK1 (proprotein convertase 1) that was downregulated in the AD group, CPE that was upregulated in the AD group, and CTSH (cathepsin H) which was absent in the AD group compared to age-matched controls. With respect to proteases known to cleave the tau protein,^19,55^ CAPN2 (calpain 2) was upregulated in AD compared to controls in this study. These findings show differential protease expression that may participate in production of synaptic neuropeptides and tau peptides in AD compared to control brain cortex.

In addition, our results showing upregulation or downregulation of neuropeptides and tau peptides in AD synaptosomes may consider the change in peptide levels to result from peptide biosynthesis combined with release of these peptides at the synapse. The synaptic nerve terminal has the essential functions of neurotransmitter biosynthesis and release. It will be of interest for future studies to assess both the neurotransmitter metabolism and release mechanisms.

The findings of altered neuropeptide and tau peptide signatures in AD synaptosomes were subjected to rigorous statistical evaluation for the four biological samples for each AD and control group. While a small sample size was used in this study, the significant statistical evaluations indicate that the results are significant. It will be important in future studies to utilize larger sample group sizes to assess the hypothesis for alterations in neuropeptide and tau peptide signatures in human AD brain regions during progression of the disease.

Future investigations of synaptic neurotransmitter functions and structures of synapses in AD brains will be important to gain an in-depth understanding of neurodegeneration in AD. The synaptosome preparation provides a model of the *in vivo* status of synapses in human AD brains. Synaptosomes retain the ability to release neurotransmitters for a period of about 1 day postmortem,^20,23^ indicating the integrity of neurotransmitter secretory vesicles and synaptic release mechanisms. Synaptosomal preparations contain presynaptic and postsynaptic structures, and the portion of such nerve terminals with or without postsynaptic features varies. It will be important for future studies to investigate the morphology of synaptosomes and their functional neurotransmitter signatures from different brain areas during early to late stages of AD during progressive neurodegeneration involving amyloid plaques and neurofibrillary tangle pathology.

Overall, this study demonstrates differential signatures of neuropeptides and tau peptides identified in AD compared to control human brain cortex synaptosomes. These findings indicate dysregulation of synaptic peptidergic components utilized for cell–cell communication in AD by neuropeptides and dysregulation of the spectrum of synaptic tau peptides in AD compared to controls. These results were obtained from brain cortex samples from human AD subjects that were cognitively deficient compared to normal controls, assessed by the MMSE cognitive measure scores. It will be of interest to assess the signatures of neuropeptides and tau peptides at early to late stages of AD to evaluate synaptic peptidergic signaling components at mild to severe stages of AD.

## Experimental Methods

### AD and Age-Matched Human Brain Tissues

Human brain AD and age-matched control brain cortex tissues (temporal cortex), fresh frozen, were acquired from the Shiley-Marcos AD Neuropathology Core at UC San Diego, collected according to IRB-approved protocols. Four patients had diagnosis of AD dementia and advanced AD neuropathology postmortem (Braak stages V–VI), with an average age of 83 ± 5 (two males and two females). Four control age-matched non-demented, cognitively normal subjects lacked AD neuropathology, with an average age of control of 85 ± 6 (two males and two females). Individuals with diagnoses of diabetes or other potentially confounding conditions such as stroke were not included. Determination of AD dementia was made using the MMSE scores of cognitive functions which were available for subjects from which these human brain samples were obtained. All brain samples were de-identified and provided blinded for this investigation.

### Synaptosome Preparation from Human Brain Cortex

Synaptosomes were isolated by homogenization and differential centrifugation according to previously published methods.^20,21^ Briefly, the tissue (0.75 g) was thawed in 0.32 M sucrose in 0.1 M phosphate, pH 7.4 (sucrose-phosphate buffer), at 10% w/v at 37 °C for 2 min, and then 12.5 mL of ice-cold sucrose-phosphate buffer was added. Homogenates were prepared in a glass-Teflon homogenizer with an internal diameter of 15.9 mm and a clearance of 0.13 mm (Thomas Scientific, Philadelphia, PA) at 900 rpm. Synaptosomes were isolated by differential centrifugation at 1000*g* for 5 min, and the resultant supernatant containing synaptosomes was centrifuged at 12,000*g* for 20 min to pellet synaptosomes. Synaptosomes were resuspended in sucrose-phosphate buffer for peptidomics sample processing. The protein content of synaptosome preparations was measured by the BioRad DC protein assay (BioRad, Hercules, CA), and 1.5 mg per synaptosome sample was used for peptidomics.

### Isolation of Endogenous Low-MW Peptides

Peptides were extracted from synaptosomes by bringing the samples to 20 mM HCl (pH 3), incubation on ice for 15 min, centrifugation (14,000*g* for 30 min), and collection of the supernatant containing peptides. This peptide extract was then filtered through a 10 kDa molecular weight cutoff (MWCO) membrane (Millipore, Burlington, CA) by centrifugation (14,000*g* for 60 min), including rinsing the membrane with 0.5 M NaCl and 10 mM HCl with a second centrifugation. Filtrates from the two centrifugation steps through the 10 kDa MWCO membrane were combined and neutralized with 1 M ammonium bicarbonate to ∼30 mM. Peptide concentration was determined using a Pierce Quantitative Colorimetric Peptide Assay Kit (Thermo Fisher).

### Peptidomics–Neuropeptidomics Analysis

Reduction of the low-MW peptides was achieved by incubation in 6 M urea-Tris and 100 mM dithiothreitol (DTT) (60 min), followed by alkylation in iodoacetamide (17.6 mM, 30 min incubation at room temperature) with quenching by addition of DTT to 4.46 mM. Peptide samples were acidified with to 0.1% trifluoroacetic acid (TFA) for desalting using solid-phase extraction (SPE) (StrataX, Phenomenex, Torrance, CA) as has been described.^7,45^ Peptide samples were dried in a vacuum centrifuge and resuspended in 2% acetonitrile (ACN) and 0.1% TFA at 0.5 μg/μL for nano-LC–MS/MS using 500 ng per injection (two technical replicate injections per sample).

Nano-LC–MS/MS tandem mass spectrometry was performed using a Dionex UltiMate 3000 nano liquid chromatography unit and a hybrid quadrupole/orbitrap Q-exactive mass spectrometer (Thermo Fisher Scientific). Samples were injected at a randomized order at a flow rate of 275 nL/min on an 80 min gradient of 5 to 40% ACN with 0.1% formic acid, followed by a 20 min gradient of 85 to 90% ACN with 0.1% formic acid (using buffer A of 0.1% formic acid in H_2_O and buffer B of ACN with 0.1% formic acid). The column contained ethylene-bridged hybrid (BEH) C18 of 1.7 μm diameter heated to 65 °C. Data-dependent acquisition of mass spectra was obtained in the positive ion mode. MS1 microscans were acquired for scan range 310–1200 *m*/*z* at a resolution of 70,000 at 200 *m*/*z* and an injection time of 50 ms at an AGC target of 3 × 10^6^. Data-dependent MS2 was acquired in a 1.5 *m*/*z* isolation window at a resolution of 17,500, a scan range of 200–2000 *m*/*z*, a fixed first mass of 150 *m*/*z*, a maximum inject time of 50 ms, an automatic gain target of 1 × 10^5^, an intensity threshold of 1 × 10^4^, an HCD cell normalized collision energy of 27 V, and a dynamic exclusion of 20 s. The LC–MS report for peptidomics is provided as the Supporting Information (data Supplement S1).

Bioinformatics of peptidomics mass spectrometry data was conducted as has been previously described^7^ and summarized here. Peptidomics data were subjected to protein precursor identification, and LFQ was conducted using PEAKS studio 8.5 (Bioinformatics Solutions, Inc., Waterloo, ON, Canada) using the complete human protein sequence database (UniprotKB/SwissProt) for peptide sequence searches. Peptides were considered identified in a biological replicate if it was present in one of two technical replicates and considered present in a biological sample group if it was present in three out of four biological brain samples. The peptidomics PEAKS parameters and data output are provided in Supporting Information data (data Supporting Informaton S2 and data Supporting Information S3).

Neuropeptidomics data were obtained by identification of neuropeptides of the peptidomics data set using the NeuroPedia database of all known neuropeptides.^25^ Neuropeptidomics data were compiled (data Supporting Information S4). Peptides from AD and control groups were mapped onto precursor protein sequences obtained from Uniprot.

### Cleavage Site Analysis of Neuropeptides within Proneuropeptide Sequences

Proteolytic cleavage site analysis of neuropeptides derived from proneuropeptide precursors was assessed by *z*-scores and iceLogo evaluations, conducted as has been previously reported.^7,50^ These assessments calculated the frequencies of amino acid residues at the P1–P1′ cleavages sites and at the neighboring residues at the P4 to P4′ position adjacent to the cleaved peptide bonds. Evaluations involved *z*-scores calculated by *X* – μ/σ, where *X* is the frequency of the amino acid in the experimental data set, μ is the frequency of a particular amino acid at a specific position in the reference set (random set of amino acids as the negative data set), and σ is the standard deviation (SD of the experimental set compared to the random set). The resulting values indicated the standard deviation of the frequency of an amino acid in the experimental data set compared to the random data set. *z*-scores were utilized to generate iceLogo illustrations^50^ of the relative frequencies of residues at each of the P4 to P4′ positions of the cleavage sites. The heights of the single-letter amino acids indicate “percent difference”, representing the difference in frequency for a residue appearing in the positive data set relative to the negative data set. Positive differences are shown above the midline, and negative differences are represented below the midline.

### Proteomics Analysis

Trypsin digestion of synaptosome proteins (200 μg) was conducted for proteomics analysis. Proteins were precipitated in 90% methanol by incubation on ice for 15 min, followed by centrifugation (14,000*g* for 15 min). The protein pellet was dried, resuspended in 200 μL of sodium deoxycholate (SDC) buffer containing 1% SDC, 100 mM tris, pH 8, 40 mM 2-chloroacetaminde, and 10 mM tris(2-carboxyethyl)phosphine, and incubated at 95 °C for 10 min and then cooled to room temperature (5 min). Trypsin/LysC (Promega, Madison, WI) was added at an enzyme/protein ratio of 1:50 and incubated at 37 °C overnight, followed by quenching by addition of TFA to 0.3% (pH < 3). Samples were desalted and purified using SPE by applying peptides to Empore C18 membranes (3M, Maplewood, MN), washing with 0.1% TFA, and eluted with ACN/0.1% TFA, as described previously.^50^ Peptide concentrations were measured using a Pierce Total Peptide Assay kit (ThermoFisher). Samples were dried in a vacuum centrifuge and resuspended in 2% ACN and 0.1% TFA at a peptide concentration of 0.5 μg/μL. 2 μg per sample was used for nano-LC–MS/MS.

Nano-LC–MS/MS was conducted using a Dionex UltiMate 3000 nano-LC and an Orbitrap Q-exactive (Thermo Fisher) for tandem mass spectrometry. Samples were injected (two technical replicates per sample) in a randomized order at a flow rate of 300 nL/min using a 180 min gradient of 5–25% ACN in 0.1% formic acid, followed by a 20 min gradient of 85–90% ACN in 0.1% formic acid. The LC column contained ethylene-bridged hybrid C18 of a 1.7 μm diameter column heated to 65 °C. Mass spectra were acquired in the positive ion mode with a full data-dependent MS scan. MS1 microscans were acquired for a scan range of 310–1200 *m*/*z* at a resolution of 70,000 at 200 *m*/*z* and an injection time of 100 ms. Data-dependent MS2 was acquired in a 1.5 *m*/*z* isolation window at resolution of 17,500, a maximum inject time of 50 ms, an automatic gain target of 1 × 10^5^, an intensity threshold of 4 × 10^3^, and an HCD cell normalized collision energy of 27 V. The LC–MS/MS report of parameters is provided in the Supporting Information (data Supplement S5).

Protein identification utilized PEAKS (v. 8.5) bioinformatics analysis of mass spectrometry data using the decoy-fusion method. Mass spectra were searched against the UniprotKB/SwissProt human protein database containing 71,783 entries. PTMs searched were carbamidomethylation on Cys, oxidation of Met, N-terminal acetylation, and phosphorylation at Ser, Thr, and Tyr, with a maximum of 3 PTMs searched per precursor. The PTM Ascore of local confidence was set to ≥13, which corresponds to approximate *p* < 0.02. The monoisotopic precursor mass error tolerance was 20 ppm with a fragment mass error tolerance of 0.01 Da. Identification parameters resulted in a false discovery rate (FDR) of 0.5% with peptide identification of −log_10_(*P*) ≥ 32 and protein identification of −log_10_(*P*) ≥ 55. Further technical details of protein identification PEAKS search are provided in data Supporting Information S6. Precursor ions and assigned protein identifications are provided in data Supporting Information S7. Summaries of protein groups with LFQ are provided in data Supporting Information S8 (master table), including the coverage and number of spectra in each sample and listing proteins of AD and control groups.

LFQ of proteins was achieved by PEAKS analysis (v. 8.5) whereby peptide features of the MS1 charge states were converted to area under the curve (AUC) and summed to determine peptide AUC. This quantitation was based on peptide features of precursor mass, peak height, intensity, isotope pattern, and retention time via extracted ion chromatographs. To determine relative protein abundance, AUCs of the peptides representing the protein were summed. Assignment of a protein as present in each of the AD or control groups required that the protein was present in at least three out of four biological replicate samples.

Quality parameters for LFQ determination were set as peptide quality >0.3 and an abundance of 1 × 10^4^. The AUCs of MS1 charge state peptides of technical replicate samples were included for LFQ if eluted within 3 min and peptide features (listed in the previous paragraph) matched. Modifications were excluded for LFQ. LOESS-G was used as a normalization method for LFQ intensity distributions using the Normalyzer web application as has been previously described.^22^ Technical replicate reliability was restricted by −log_10_(*P*), with quality assessment as 1/log(σ), where σ is the technical variance between samples. LFQ MS1 peak areas of each peptide are associated with peptide de novo assisted database identification by MS2 peptide feature mapping.

Protein groups with 0 intensity value were imputed with a random value within the lowest 5% of values within a standard deviation of 1 of the distribution of all protein intensity values. Protein isoforms were inspected to assure that the same LFQ values were assigned to isoforms of a protein group. To determine quantifiable proteins that were significantly different between AD and controls, biological replicates were averaged and Student’s *t*-test was used to determine *p* values which were considered significantly different at *p* < 0.05. Bioinformatics analyses containing identified proteins and the set of identified proteins that were also quantifiable are summarized in the master table (data Supporting Information S8).

Protease components of proteomics data were identified using the MEROPS protease database.^30^ Proteases in the AD and control groups were compared for those present in only the AD group, present in only the control group, and present in both groups with upregulation and downregulation.

Upregulation and downregulation of proteins shared in AD and control groups were assessed by ratios of log_2_(AD/control) intensities of protein levels at significance levels of *p* < 0.05 (by Student’s *t*-test). Heatmap illustration of significantly upregulated and downregulated proteases was generated using pheatmap in R studio using Euclidean hierarchical clustering with complete linkage (https://www.rstudio.com/products/rstudio/.^56^

### Protein Interactions Predicted by STRING and GO Bioinformatics Analysis

Selected groups of proteins were assessed for predicted protein interaction networks by STRING and GO analysis^27,28^ by mining databases of known protein interactions (DIP, BioGRID, HPR, IntAct, MINT, PDB, and others). Protein–protein interactions were considered significant if an interaction probability was more likely in these data than a random group of interactions of proteins of the same number at a high confidence score of 0.7. GO enrichment was determined with using STRING-db. Enrichment was determined to be significant if FDR <1% using Benjamini–Hochberg hypergeometric probability testing procedures that determine the statistical probability of protein being present in a GO term compared to the total genes in the GO pathway.^57,58^

## Supporting Information Available

Peptide mapping to proneuropeptides of CHGB, SCG2, SCG3, CCK, and SST; peptides in AD and controls; and neuropeptide counts in AD and controls (PDF)
Peptidomics–neuropeptidomics for the LC–MS report (PDF)
Peptidomics PEAKS report (PDF)
Peptides and neuropeptides (XLSX)
Neuropeptidomics master table (XLSX)
LC–MS report for synaptosome proteomics (PDF)
Proteomics PEAKS report (PDF)
Proteomics peptides and proteins (XLSX)
Proteomics and proteomics master table (XLSX)

## Notes

For peptidomics–neuropeptidomics data, LC–MS/MS files can be accessed through www.proteomexchange.org with identifier number PXD032779 and through www.massive.ucsd.edu with identifier number MSV000089121. For proteomics data, LC–MS/MS files can be accessed through www.proteomexchange with identified number PXD032805 and through www.massive.ucsd.edu with identifier number MSV000089126.
