# Supplementary material for: Dysregulation of Neuropeptide and Tau Peptide Signatures in Human Alzheimer’s Disease Brain
Source: ACS Chem Neurosci. 2022 Jun 27;13(13):1992–2005. doi: 10.1021/acschemneuro.2c00222 (PMC9264367; doi:10.1021/acschemneuro.2c00222)
Supplement: Supplementary file 1 — cn2c00222_si_001.pdf [file cn2c00222_si_001.pdf]

Supplemental Information

Manuscript Title:
Dysregulation of Neuropeptide and Tau Peptide Signatures in Human Alzheimer's Disease Brain

Authors:
Sonia Podvin[1], Zhenze Jiang[1], Ben Boyarko[1], Leigh-Ana Rossitto[2], Anthony O'Donoghue[1], Robert A. Rissman[3], and Vivian Hook [1,2]*

Affiliations:
[1]Skaggs School of Pharmacy and Pharmaceutical Sciences, University of California, San Diego, La Jolla, CA;
[2]Biomedical Sciences Graduate Program, University of California, San Diego, La Jolla, CA;
[3]Department of Neurosciences, University of California San Diego, La Jolla, CA

Supplemental Information (SI) Figures:

SI Figure S1. CHGB (secretogranin 1) peptide map.

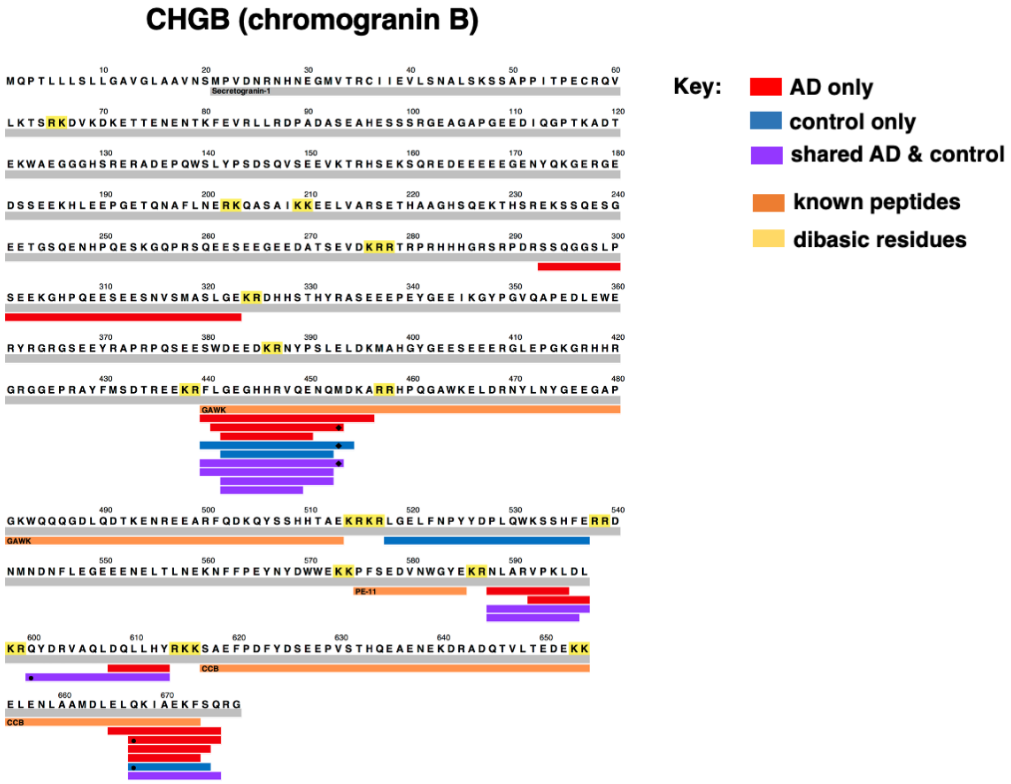

SI Figure S2. Secretogranin 2 (SCG2) peptide map.

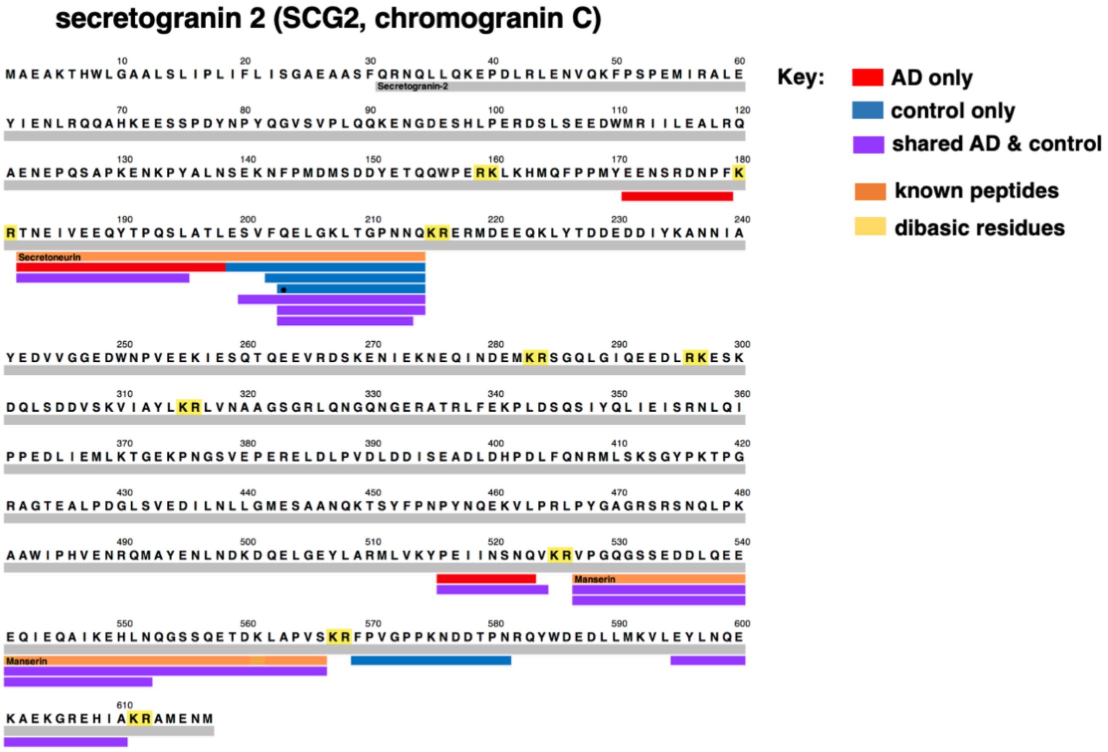

SI Figure S3. Secretogranin 3 (SCG3) peptide map.

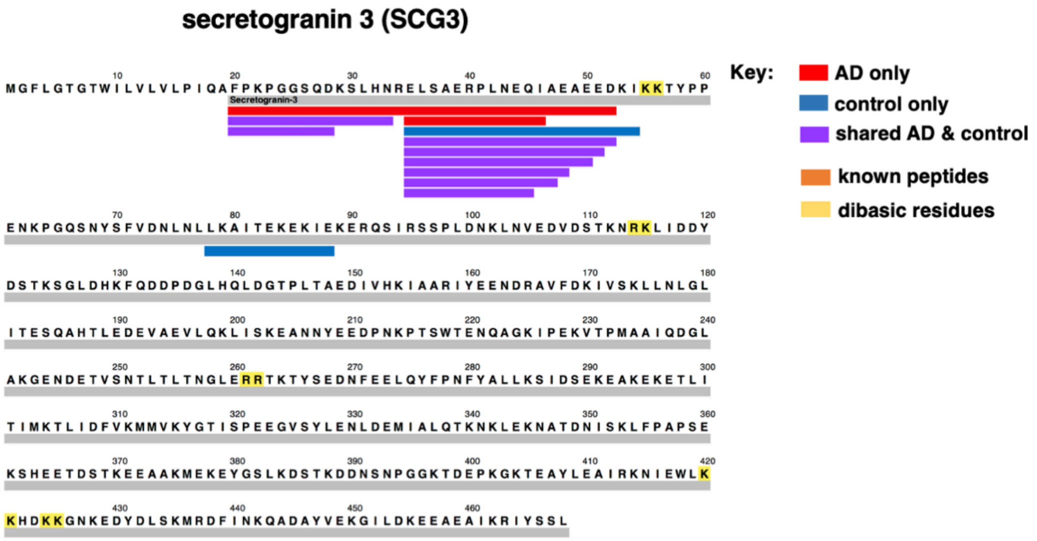

SI Figure S4. Cholecystokinin (CCK) peptide map.

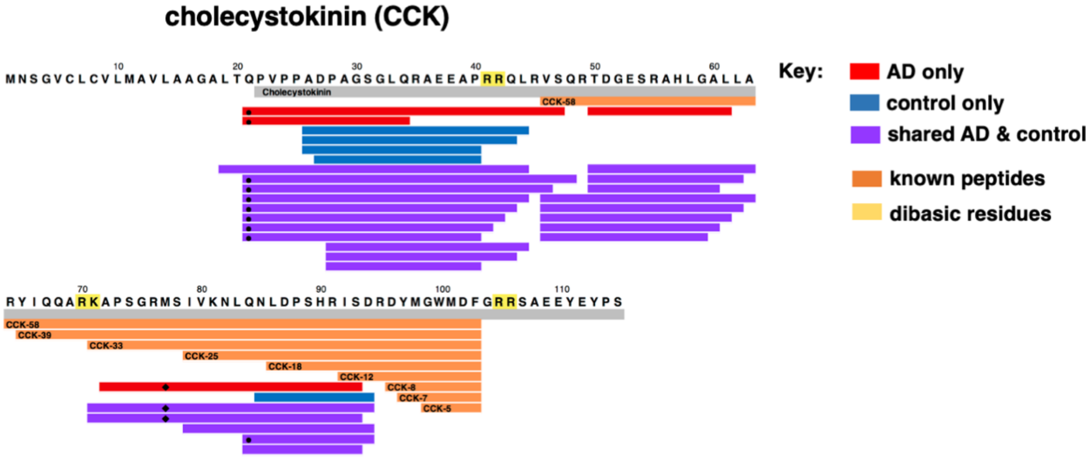

SI Figure S5. Somatostatin (SST) peptide map.

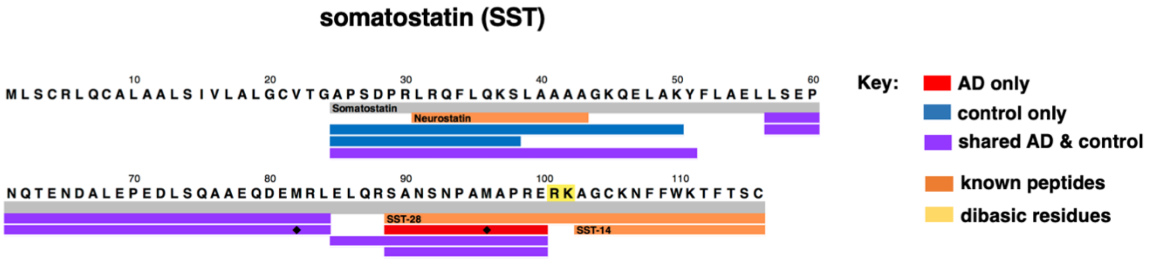

SI Figure S6. Peptidomics in AD and control synaptosomes

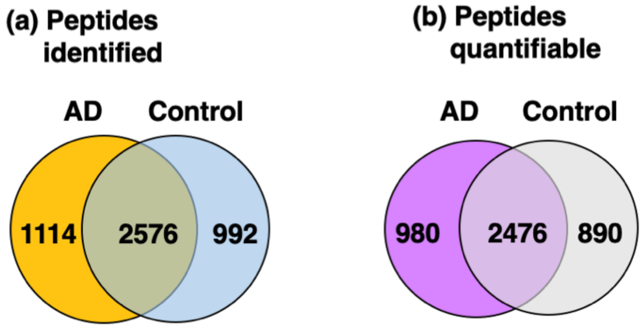

SI Table:

Supplemental Table S1. Number of Neuropeptides Derived from Proneuropeptides

Proneuropeptide	All Peptides	AD Total Peptides	AD Only Peptides	Control Total Peptides	Control Only Peptides	Shared Peptides
CARTPT	1	1	1	0	0	0
CBLN4	1	1	0	1	0	1
CCK	34	29	3	31	5	26
CHGA	22	20	11	11	2	9
CHGB	23	19	11	12	4	8
PENK	2	2	0	2	0	2
SCG2	15	11	3	12	4	8
SCG3	12	10	2	10	2	8
SCG5	3	2	0	3	1	2
SST	8	6	1	7	2	5
TAC3	3	2	1	1	0	1
VGF	34	11	0	34	23	11
VIP	1	0	0	1	1	0

The number of neuropeptides derived from each proneuropeptide precursor is shown according the indicate categories of the columns.
