# Supplementary material for: Dysregulation of Neuropeptide and Tau Peptide Signatures in Human Alzheimer’s Disease Brain
Source: ACS Chem Neurosci. 2022 Jun 27;13(13):1992–2005. doi: 10.1021/acschemneuro.2c00222 (PMC9264367; doi:10.1021/acschemneuro.2c00222)
Supplement: Supplementary file 2 — cn2c00222_si_002.pdf [file cn2c00222_si_002.pdf]

Supplement 1 - LC- MS/MS report for synaptosome peptidomics

Samples were resuspended in 2% ACN, 0.1% TFA to a total peptide concentration of 130 ng/uL

Each sample was injected and analyzed twice, 520 ng total peptide per injection
Column was 25 cm long, heated to 65 °C
Solvent A - water, 0.1% formic acid
Solvent B - acetonitrile, 0.1% formic acid

Method of Q Exactive

Overall method settings

**Global Settings**	
Use lock masses	off
Lock mass injection	—
Chrom. peak width (FWHM)	15 s
**Time**	
Method duration	115.00 min
**Customized Tolerances (+/-)**	
Lock Masses	—
Inclusion	—
Exclusion	—
Neutral Loss	—
Mass Tags	—
Dynamic Exclusion	10.0 ppm

Experiment

Full MS / dd-MS² (TopN)

**General**	
Runtime	0 to 95 min
Polarity	Positive
In-source CID	0.0 eV
Default charge state	2
Inclusion	—
Exclusion	—
Tags	—
**Full MS**	
Microscans	1
Resolution	70,000
AGC target	3e6
Maximum IT	100 ms
Number of scan ranges	1
Scan range	310 to 1250 m/z
Spectrum data type	Profile
**dd-MS² / dd-SIM**	
Microscans	1
Resolution	17,500
AGC target	1e5
Maximum IT	50 ms
Loop count	10
MSX count	1

TopN	10
Isolation window	1.5 m/z
Isolation offset	0.0 m/z
Scan range	200 to 2000 m/z
Fixed first mass	150.0 m/z
(N)CE / stepped (N)CE	nce: 27
Spectrum data type	Centroid
**dd Settings**	
Minimum AGC target	5.00e2
Intensity threshold	1.0e4
Apex trigger	—
Charge exclusion	unassigned, 1, 8, >8
Peptide match	Preferred
Exclude isotopes	on
Dynamic exclusion	20.0 s
If idle ..	do not pick others

Setup

Tunefiles

General

Switch Count 0

Base Tunefile C:\Xcalibur\methods\CL_nanoTune_20180409.mstune

Contact Closure

General

Used False

Start in Closed True

LC gradient

Solvent A - water, 0.1% formic acid

Solvent B - acetonitrile, 0.1% formic acid

Time	Flow (ul/min)	%A	%B
0.000	0.300	99	1.0
0.100	0.275	95	5.0
75.000	0.275	75	25.0
75.100	0.275	70	30.0
80.000	0.275	60	40.0
85.000	0.275	15	85.0
95.000	0.275	15	85.0
95.100	0.400	10	90.0
105.000	0.400	10	90.0
105.100	0.300	99	1.0
115.000	Stop Run		
