# Supplementary material for: Dysregulation of Neuropeptide and Tau Peptide Signatures in Human Alzheimer’s Disease Brain
Source: ACS Chem Neurosci. 2022 Jun 27;13(13):1992–2005. doi: 10.1021/acschemneuro.2c00222 (PMC9264367; doi:10.1021/acschemneuro.2c00222)
Supplement: Supplementary file 3 — cn2c00222_si_003.pdf [file cn2c00222_si_003.pdf]

1. Notes

2. Result Statistics

Figure 1. False discovery rate (FDR) curve. X axis is the number of peptide-spectrum matches (PSM) being kept. Y axis is the corresponding FDR.

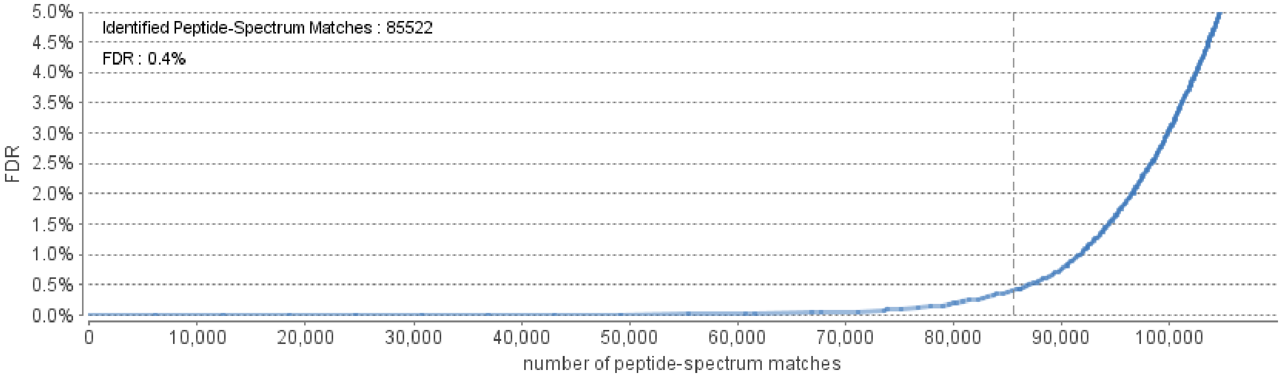

Figure 2. PSM score distribution. **(a)** Distribution of PEAKS peptide score; **(b)** Scatterplot of PEAKS peptide score versus precursor mass error.

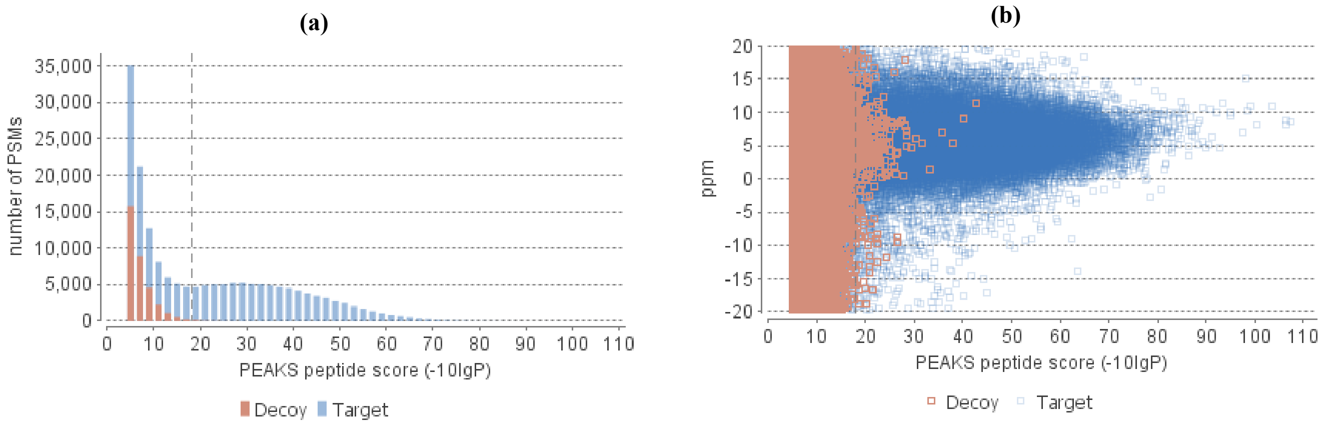

Figure 3. De novo result validation. Distribution of residue local confidence: **(a)** Residues in de novo sequences validated by confident database peptide assignment; **(b)** Residues in "de novo only" sequences.

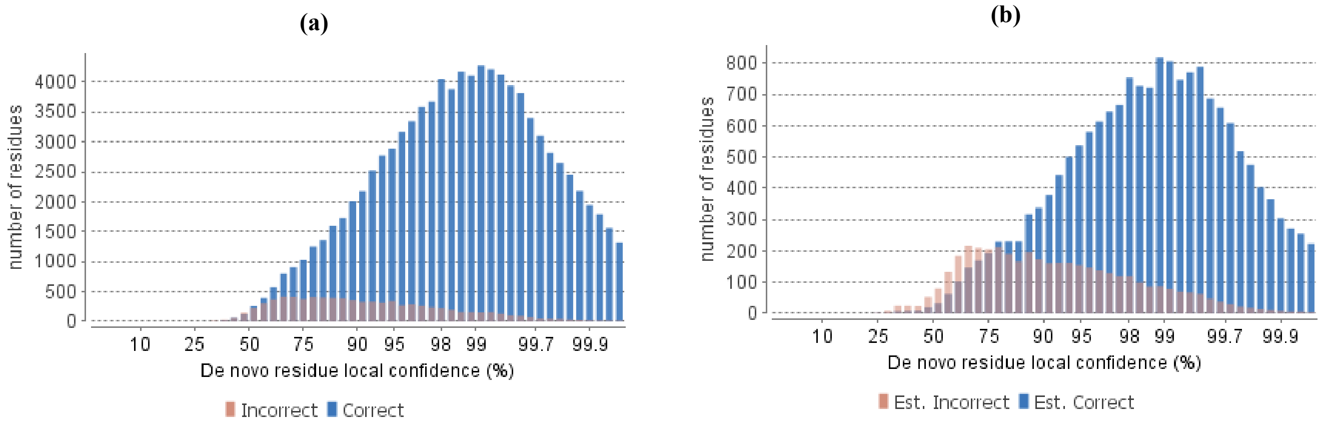

Table 1. Statistics of data.

# of MS scans	107690
# of MS/MS scans	666444

Table 4. PTM profile.

Name	ΔMass	Position	#PSM	-10lgP	Area	AScoı

Oxidation	15.99	M	10654	81.85	1.26E7	1000.(
Acetylation	42.01	Protein N-term	9822	89.25	2.16E5	1000.(
Pyro-glu from Q	-17.03	N-term	986	83.45	1E6	1000.(
Amidation	-.98	C-term	531	58.91	1.57E6	1000.(

Table 2. Result filtration parameters.

Peptide -10lgP	≥18
Peptide Ascore	≥0
Protein -10lgP	≥29
Proteins unique peptides	≥1
De novo ALC Score	≥90%

Table 3. Statistics of filtered result.

Peptide-Spectrum Matches	85522
Peptide sequences	16146
Protein groups	1307
Proteins	1331
Proteins (#Unique Peptides)	740 (>2); 205 (=2); 316 (=1);
FDR (Peptide-Spectrum Matches)	0.4%
FDR (Peptide Sequences)	1.4%
FDR (Protein)	5.6%
De Novo Only Spectra	2085

3. Experiment Control

Figure 4. Precursor mass error of peptide-spectrum matches (PSM) in filtered result. **(a)** Distribution of precursor mass error in ppm; **(b)** Scatterplot of precursor m/z versus precursor mass error in ppm.

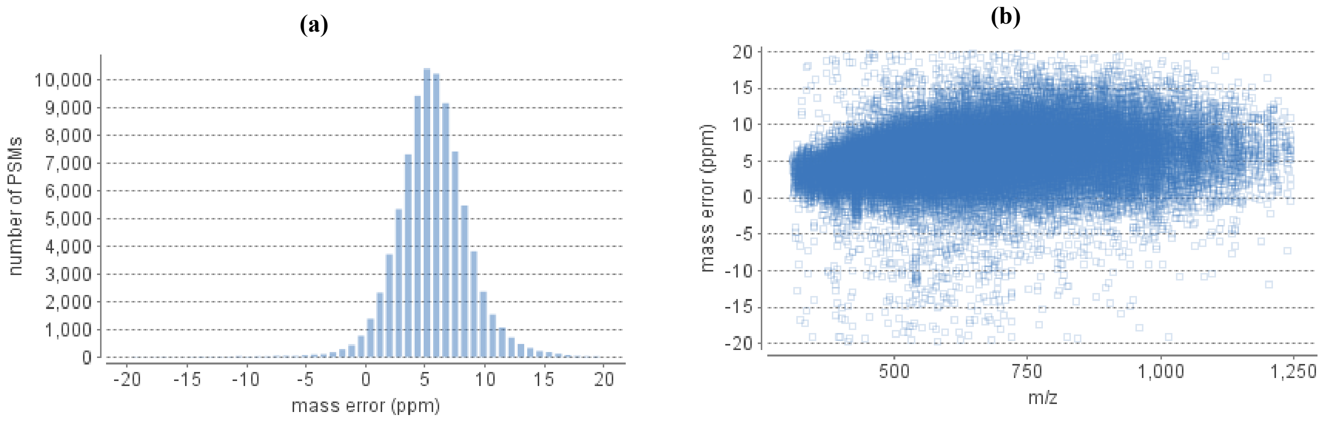

Table 5. Number of identified peptides in each sample by the number of missed cleavages

Missed Cleavages	0	1	2	3	4+
C1_1	0	0	0	0	1683
C1_2	0	0	0	0	544
C2_1	0	0	0	0	716
C2_2	0	0	0	0	654
C3_1	0	0	0	0	980
C3_2	0	0	0	0	1387
C4_1	0	0	0	0	357
C4_2	0	0	0	0	397
AD1_1	0	0	0	0	1686
AD1_2	0	0	0	0	1302
...					

4. Other Information

Table 6. Search parameters.
Search Engine Name: PEAKS

Table 7. Instrument parameters.
Fractions: SP_20180810_SynaptoPep_C1_1.raw, SP_20180810_Synapto

Parent Mass Error Tolerance: 20.0 ppm
Fragment Mass Error Tolerance: 0.01 Da
Precursor Mass Search Type: monoisotopic
Enzyme: None
Max Missed Cleavages: 100
Non-specific Cleavage: both
Variable Modifications:
Oxidation (M): 15.99
Acetylation (Protein N-term): 42.01
Pyro-glu from Q: -17.03
Amidation: -0.98
Max Variable PTM Per Peptide: 3
Database: Hsapiens_SP_20180416
Taxon: All
Searched Entry: 20301
FDR Estimation: Enabled
Different data refine parameters are used for this search:

Pep_C1_2.raw, SP_20180810_SynaptoPep_C2_1.raw, SP_20180810_SynaptoPep_C2_2.raw, SP_20180810_SynaptoPep_C3_1.raw, SP_20180810_SynaptoPep_C3_2.raw, SP_20180810_SynaptoPep_C4_1.raw, SP_20180810_SynaptoPep_C4_2.raw, SP_20180810_SynaptoPep_AD1_1.raw, SP_20180810_SynaptoPep_AD1_2.raw, SP_20180810_SynaptoPep_AD2_1.raw, SP_20180810_SynaptoPep_AD2_2.raw, SP_20180810_SynaptoPep_AD3_1.raw, SP_20180810_SynaptoPep_AD3_2.raw, SP_20180810_SynaptoPep_AD4_1.raw, SP_20180810_SynaptoPep_AD4_2.raw
Ion Source: ESI(nano-spray)
Fragmentation Mode: high energy CID (y and b ions)
MS Scan Mode: FT-ICR/Orbitrap
MS/MS Scan Mode: FT-ICR/Orbitrap
