# Supplementary material for: Dysregulation of Neuropeptide and Tau Peptide Signatures in Human Alzheimer’s Disease Brain
Source: ACS Chem Neurosci. 2022 Jun 27;13(13):1992–2005. doi: 10.1021/acschemneuro.2c00222 (PMC9264367; doi:10.1021/acschemneuro.2c00222)
Supplement: Supplementary file 7 — cn2c00222_si_007.pdf [file cn2c00222_si_007.pdf]

1. Notes

2. Result Statistics

Figure 1. False discovery rate (FDR) curve. X axis is the number of peptide-spectrum matches (PSM) being kept. Y axis is the corresponding FDR.

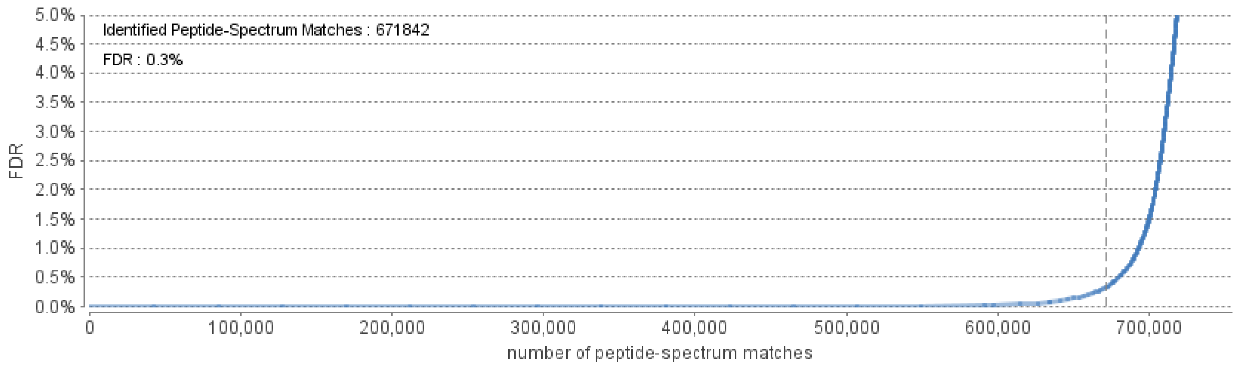

Figure 2. PSM score distribution. **(a)** Distribution of PEAKS peptide score; **(b)** Scatterplot of PEAKS peptide score versus precursor mass error.

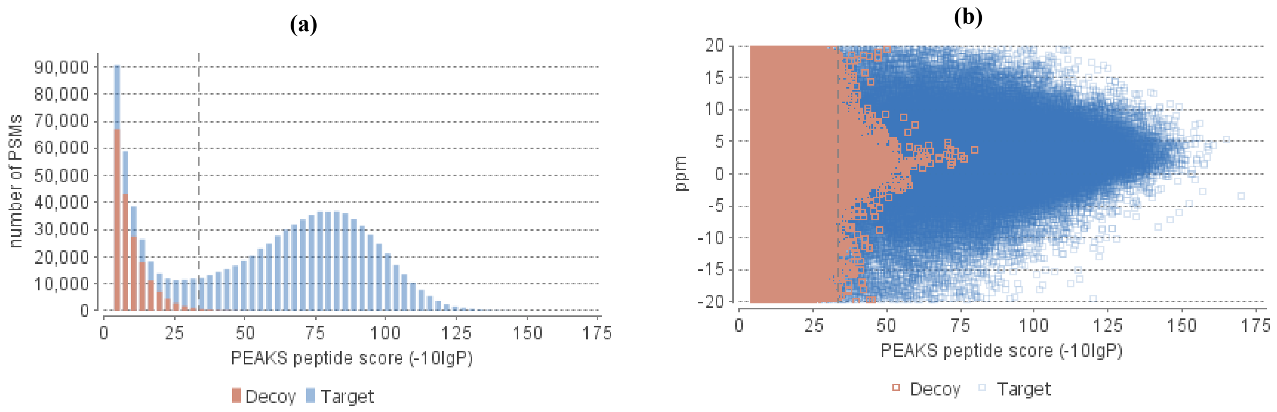

Figure 3. De novo result validation. Distribution of residue local confidence: **(a)** Residues in de novo sequences validated by confident database peptide assignment; **(b)** Residues in "de novo only" sequences.

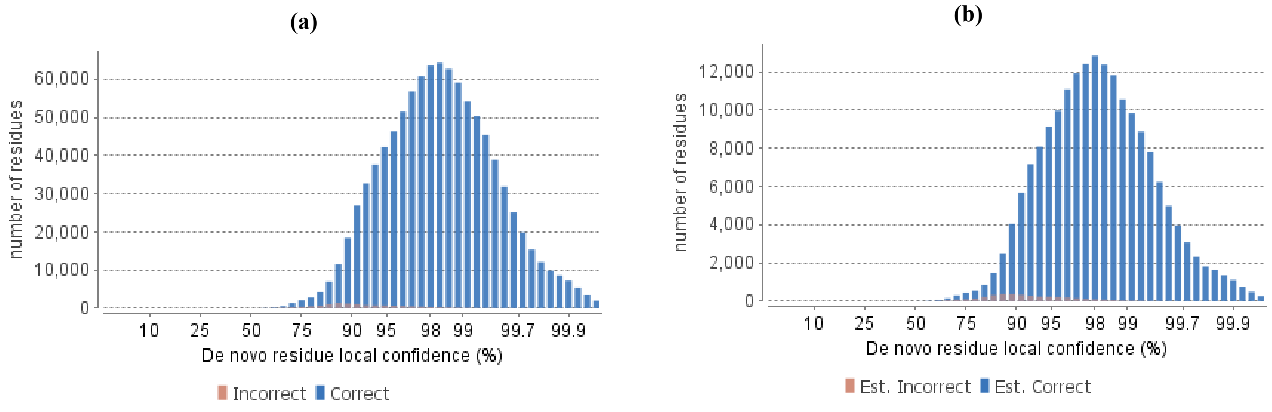

Table 1. Statistics of data.

# of MS scans	123606
# of MS/MS scans	1392151

Table 2. Result filtration parameters.

Table 4. PTM profile.

Name	ΔMass	Position	#PSM	-10lgP	Area	AS
Carbamidomethyl	57.02	C	114974	170.17	4.97E7	10(
Oxidation	15.99	M	30378	150.24	8.49E6	10(

Peptide -10lgP	≥33.5
Peptide Ascore	≥13
Protein -10lgP	≥72.7
Proteins unique peptides	≥1
De novo ALC Score	≥95%

Acetylation	42.01	Protein N-term	8702	136.99	10[illegible]

Table 3. Statistics of filtered result.

Peptide-Spectrum Matches	670278
Peptide sequences	72322
Protein groups	5939
Proteins	10050
Proteins (#Unique Peptides)	7104 (>2); 1290 (=2); 1562 (=1);
FDR (Peptide-Spectrum Matches)	0.3%
FDR (Peptide Sequences)	0.9%
FDR (Protein)	0.9%
De Novo Only Spectra	19770

3. Experiment Control

Figure 4. Precursor mass error of peptide-spectrum matches (PSM) in filtered result. **(a)** Distribution of precursor mass error in ppm; **(b)** Scatterplot of precursor m/z versus precursor mass error in ppm.

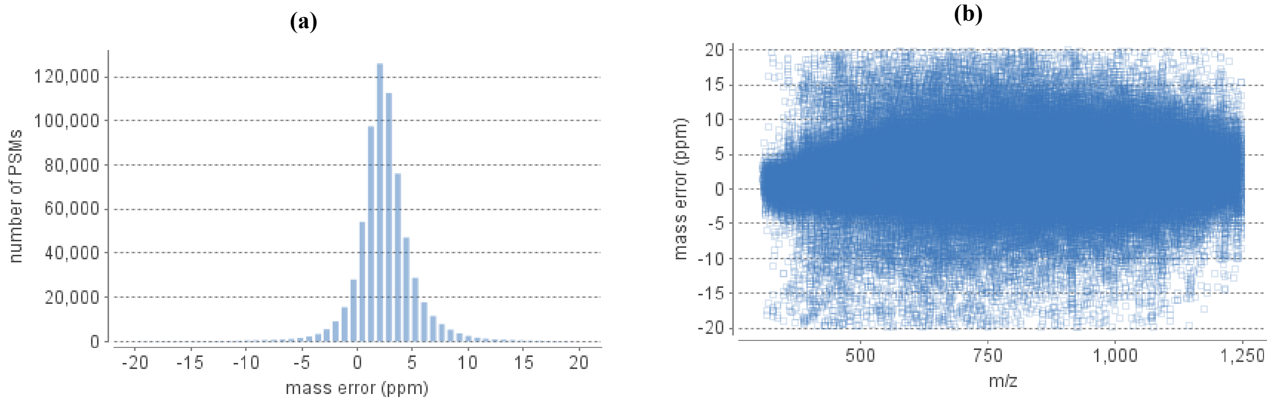

Table 5. Number of identified peptides in each sample by the number of missed cleavages

Missed Cleavages	0	1	2	3	4+
AD1_1	4923	271	63	0	0
AD1_2	3775	165	58	0	0
AD2_1	1846	140	15	0	0
AD2_2	1021	74	10	0	0
AD3_1	4264	219	15	0	0
AD3_2	4941	235	8	0	0
AD4_1	10723	598	38	0	0
AD4_2	2616	150	27	0	0
Cx1_1	6144	1539	384	0	0
Cx1_2	4667	1130	286	0	0
...					

4. Other Information

Table 6. Search parameters.
Search Engine Name: PEAKS
Parent Mass Error Tolerance: 20.0 ppm
Fragment Mass Error Tolerance: 0.01 Da
Precursor Mass Search Type: monoisotopic
Enzyme: Trypsin

Table 7. Instrument parameters.
Fractions: SP_20180712_SynaptosomeProt_AD1_1.raw, SP_20180712_SynaptosomeProt_AD1_2.raw, SP_20180712_SynaptosomeProt_AD2_1.raw, SP_20180712_SynaptosomeProt_AD2_2.raw, SP_20180712_SynaptosomeProt_AD3_1.raw, SP_20180712_SynaptosomeProt_AD3_2.raw, SP_20180712_SynaptosomeProt_AD4_1.raw, SP_20180712_Synaptosome

Max Missed Cleavages: 2
Non-specific Cleavage: none
Fixed Modifications:
  Carbamidomethylation: 57.02
Variable Modifications:
  Acetylation (Protein N-term): 42.01
  Oxidation (M): 15.99
Max Variable PTM Per Peptide: 3
Database: Human_UP_Isoforms
Taxon: All
Searched Entry: 95113
FDR Estimation: Enabled
Different data refine parameters are used for this search:

Prot_AD4_2.raw, SP_20180712_SynaptosomeProt_Cx1_1.raw, SP_20180712_SynaptosomeProt_Cx1_2.raw, SP_20180712_SynaptosomeProt_Cx2_1.raw, SP_20180712_SynaptosomeProt_Cx2_2.raw, SP_20180712_SynaptosomeProt_Cx3_1.raw, SP_20180712_SynaptosomeProt_Cx3_2.raw, SP_20180712_SynaptosomeProt_Cx4_1.raw, SP_20180712_SynaptosomeProt_Cx4_2.raw
Ion Source: ESI(nano-spray)
Fragmentation Mode: high energy CID (y and b ions)
MS Scan Mode: FT-ICR/Orbitrap
MS/MS Scan Mode: FT-ICR/Orbitrap
